# Organophosphates as Versatile Substrates in Organic Synthesis

**DOI:** 10.3390/molecules29071593

**Published:** 2024-04-02

**Authors:** Petr Oeser, Tomáš Tobrman

**Affiliations:** Department of Organic Chemistry, University of Chemistry and Technology, Prague, Technická 5, 166 28 Prague 6, Czech Republic; petr.oeser@vscht.cz

**Keywords:** organophosphates, cross-coupling reactions, alkene, synthesis

## Abstract

This review summarizes the applications of organophosphates in organic synthesis. After a brief introduction, it discusses cross-coupling reactions, including both transition-metal-catalyzed and transition-metal-free substitution reactions. Subsequently, oxidation and reduction reactions are described. In addition, this review highlights the applications of organophosphates in the synthesis of natural compounds, demonstrating their versatility and importance in modern synthetic chemistry.

## 1. Introduction

**Scheme 1:**
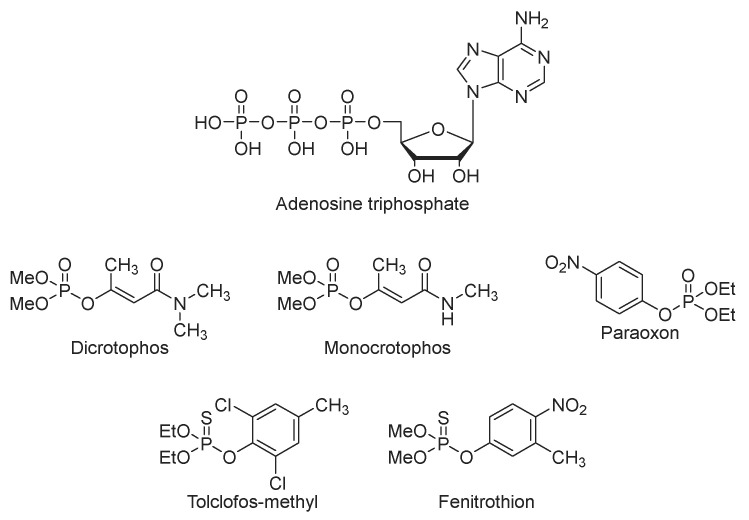
Representative examples of organophosphorus compounds related to biological systems.

Organophosphates are recognized as organic esters derived from phosphoric acid and are of significant interest because of their biological activities. A prominent example is adenosine triphosphate (ATP), which serves as a crucial energy carrier within the human body (Scheme 1). Compounds, such as dicrotophos and monocrotophos, function as acetylcholinesterase inhibitors [1] and are used as insecticides [2]. Paraoxon, a metabolic derivative of the pesticide parathion, is acknowledged as a potent cholinesterase inhibitor [3]. In addition, tochlophos-methyl is used as a fungicide [4]. Fenitrothion, used as an insecticide and known commercially as Sumithion, is an essential inclusion in the list of significant organophosphates.

Organic synthesis has experienced significant advancements in recent decades. Beyond the realm of traditional methods, techniques involving transition-metal complexes [5,6,7], electrosynthesis [8,9,10,11,12], and photochemical transformations [13,14,15] are widely used. These developments have allowed for the preparation of a wide range of compounds as well as transformations that include the formation of C–C bonds [16,17,18,19,20,21], the synthesis of heterocyclic compounds [22,23,24,25,26,27,28], and the preparation of tetrasubstituted alkenes [29,30,31,32,33,34,35,36]. In addition to conventional starting compounds, organophosphates are increasingly utilized in organic synthesis, likely due to their availability. The phosphorylation of alcohols, phenols [37], and enolates [38,39,40] represents the methods that are frequently used. Furthermore, specific reactions have been developed for the synthesis of compounds such as vinyl phosphates. The Perkow reaction [41] serves as an example, and is widely adopted for the production of vinyl phosphates.

**Scheme 2:**
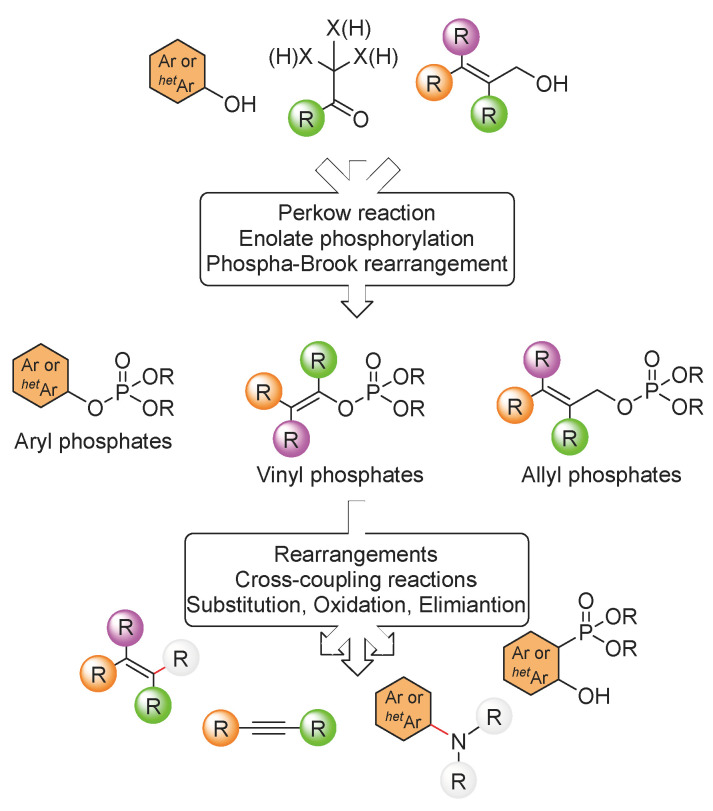
General scheme representing the synthesis and applications of organophosphates under review.

Significant interest in the chemistry of organophosphorus compounds is evident from several recent books and Special Issues in prominent journals, including *Molecules* [42,43], *Beilstein Journal of Organic Chemistry* [44], and the *Journal of Organic Chemistry* [45]. However, a comprehensive review that systematically summarizes the reactivity of organophosphates is currently missing. Therefore, the objective of this review is to summarize the main applications of organophosphates in organic synthesis over the past decade (Scheme 2). This review is structured into distinct sections for thorough coverage. The first part begins with an in-depth examination of cross-coupling reactions involving organophosphates. This section includes detailed discussion of C–H bond activations and various substitution reactions. The final part concludes with a comprehensive summary of oxidations and reductions, primarily focusing on vinyl and alkyl phosphates.

## 2. Organophosphates as Electrophiles in Transition-Metal-Catalyzed Reactions

**Scheme 3:**
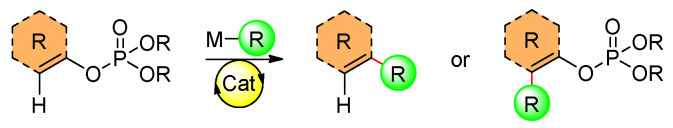
The general scheme summarizing the use of organophosphates in cross-coupling reactions.

Cross-coupling reactions frequently make use of a wide range of electrophiles. This holds true for organophosphates, which are often used due to their availability. Recent applications of organophosphates in cross-coupling reactions include the Kumada, Negishi, and Suzuki reactions. Alternatively, vinyl phosphates are employed as a directing group for the activation of the C–H bond (Scheme 3).

### 2.1. Kumada (Kumada–Tamao–Corriu) Reaction

**Scheme 4:**
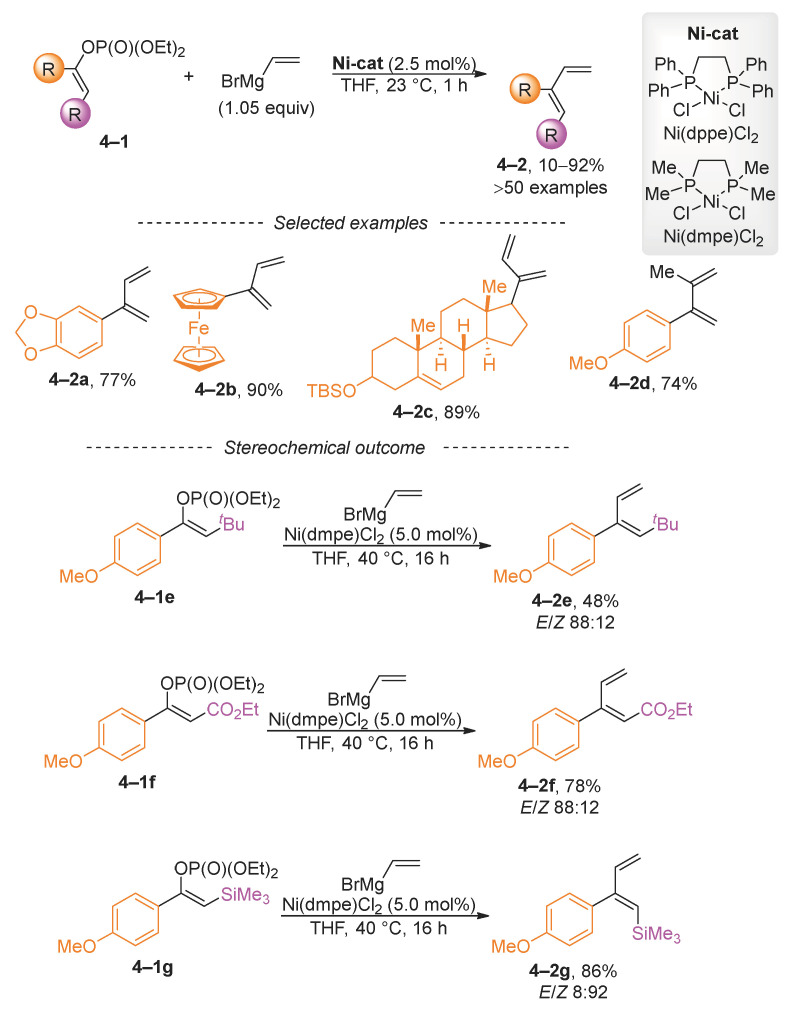
Nickel-catalyzed Kumada reaction for the synthesis of conjugated dienes.

Mazet developed a simple procedure for the preparation of conjugated dienes (Scheme 4) [46]. The process begins with diethyl vinyl phosphates **4**–**1**, which are transformed into dienes **4**–**2** through a reaction with vinylmagnesium bromide, facilitated by a catalytic amount of nickel complex. Typically conducted at room temperature, the versatility of this method is evident in a wide range of synthesized dienes. It allows for the preparation of monosubstituted dienes featuring phenyl or ferrocenyl substituents **4**–**2a** and **4**–**2b**, as well as dienes with a steroid skeleton **4**–**2c**. Although the scope has been predominantly explored using vinylmagnesium bromide, the authors also demonstrated its efficacy in generating simple disubstituted dienes, such as diene **4**–**2d**. The stereoselectivity of this cross-coupling reaction is highly influenced by the structure of the initial phosphate. For example, the reaction of phosphates **4**–**1e** and **4**–**1f** with vinylmagnesium bromide predominantly produces *E*-alkenes **4**–**2e** and **4**–**2f**. In contrast, phosphate with a trimethylsilyl group tends to undergo isomerization at the double bond, resulting in *Z*-diene **4**–**2g**. The simplicity and effectiveness of this methodology are further underscored by its widespread adoption in numerous publications [47,48,49,50,51,52,53].

**Scheme 5:**
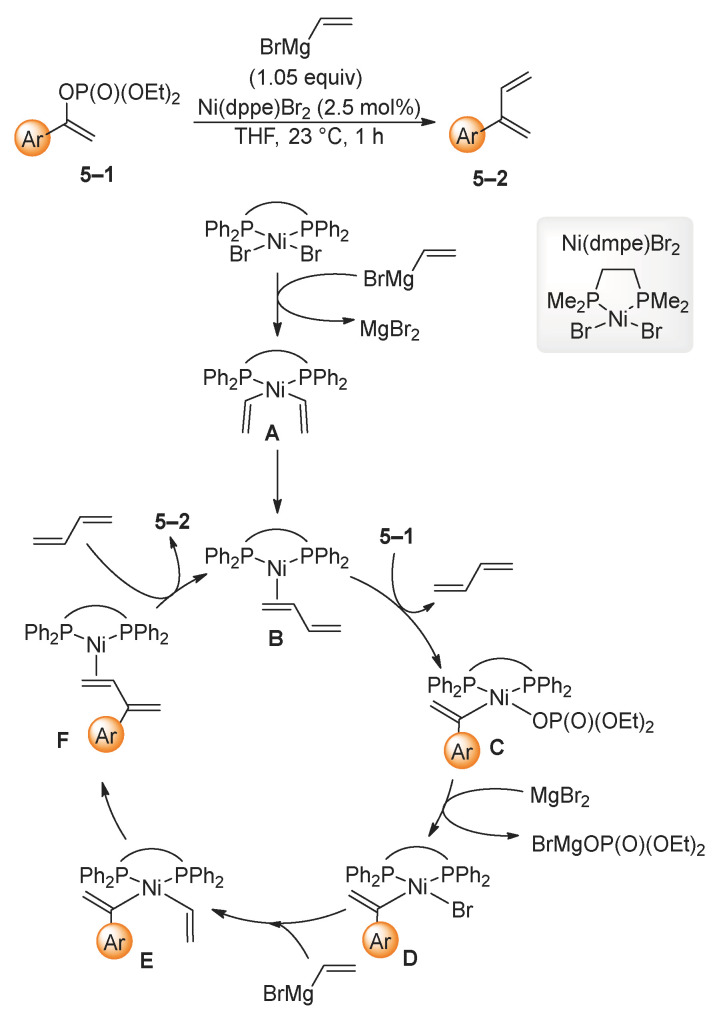
Proposed mechanism of the nickel-catalyzed Kumada coupling of vinyl diethyl phosphates.

Mazet and his team conducted an in-depth study of the mechanism underlying the Kumada reaction (Scheme 5) [54]. In this Ni(dppe)Br_2_-catalyzed reaction, active catalyst **B** is generated through a process of double transmetalation, followed by reductive elimination. Subsequently, the Ni(0) complex undergoes oxidative addition, leading to the formation of bromide complex **D** by ligand substitution. The rate-determining step of this reaction is transmetalation with vinylmagnesium bromide, which culminates in the creation of the reaction-product complex **F** via reductive elimination. A comparable mechanism is hypothesized for reactions catalyzed by the Ni(dmpe)Br_2_ complex.

**Scheme 6:**
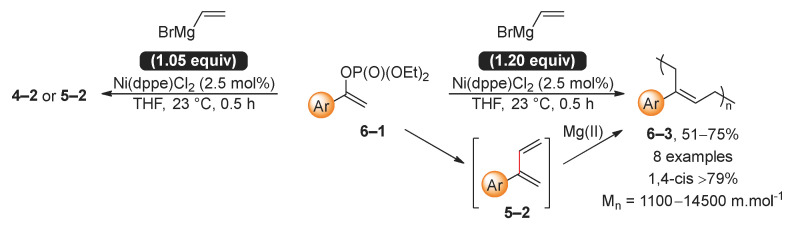
Magnesium-catalyzed polymerization as a side reaction during the Kumada reaction of vinyl phosphates.

The sensitivity of the Kumada reaction of phosphate **6**–**1** to the amount of vinylmagnesium bromide is reported (Scheme 6) [55]. When the amount of vinylmagnesium bromide exceeds 1.05 equivalence, the resultant diene **6**–**2** undergoes polymerization to produce a 1,4-*cis* polymer **6**–**3**. This polymerization process of diene **6**–**2** is facilitated by magnesium salts, as illustrated by the fact that the average molecular weight of the resultant polymer is influenced by the ratio of the starting phosphate **6**–**1** to the magnesium salt.

**Scheme 7:**
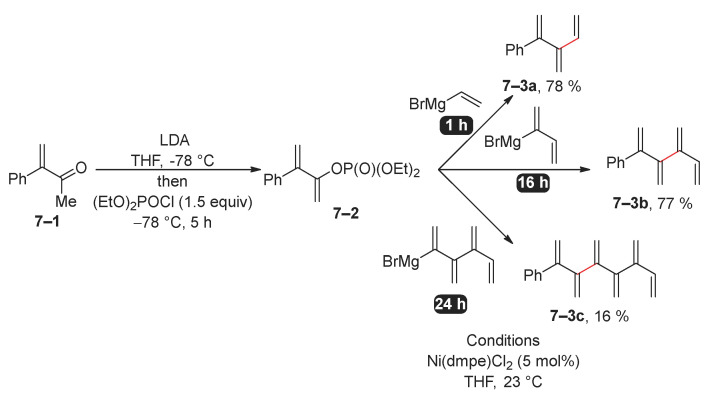
Synthesis of [n]dendralenes by the Kumada reaction of vinyl diethyl phosphates.

The same research group adopted an identical methodology to synthesize [n]dendralenes by the Kumada reaction of vinyl phosphate **7**–**2** (Scheme 7) [56]. In this process, readily available phosphate **7**–**2** was subjected to a reaction with related Grignard reagents under different conditions. In particular, [3]dendralene **7**–**3a** was efficiently synthesized using vinylmagnesium, achieving a high yield in just one hour. In contrast, the synthesis of [4]- and [6]dendralenes required significantly longer reaction times. The low yield of [6]dendralene **7**–**3c** might be attributed to the reduced stability of [n]dendralenes with an increasing number of vinyl units [57].

**Scheme 8:**
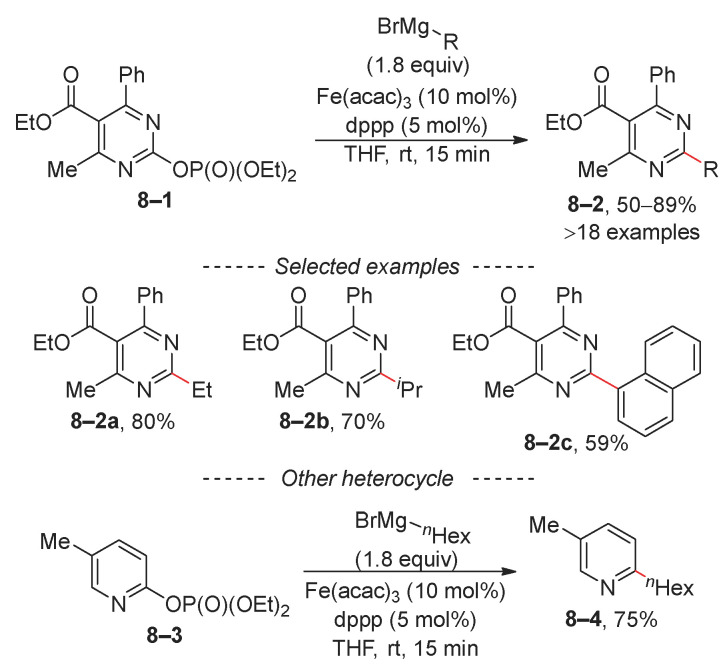
Iron-catalyzed Kumada reaction of heteroaryl diethyl phosphate.

In some cases, the Kumada reaction of organophosphates can be catalyzed by iron complexes. For example, Wang described a cross-coupling reaction of pyrimidyl diethyl phosphates with Grignard reagents that yields substituted pyrimidines **8**–**2** (Scheme 8) [58]. Although this method was successfully extended to the substituted pyridine derivative **8**–**3**, it is important to note that the reaction conditions are limited to the preparation of substituted pyrimidines **8**–**2**.

**Scheme 9:**
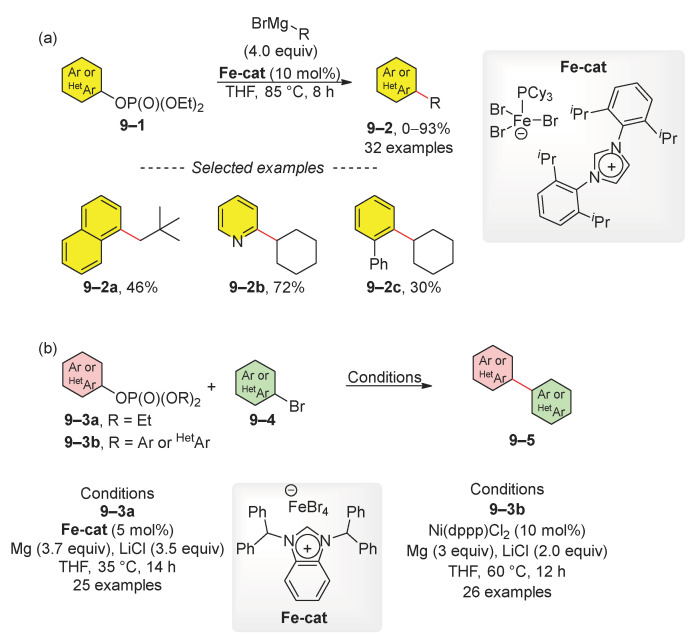
(**a**) Iron-catalyzed Kumada reaction of aryl phosphates and (**b**) iron- [60] and nickel-catalyzed [61] reductive cross-coupling reactions of aryl phosphates.

Sun and co-workers developed a novel class of ionic iron complexes. One of the prepared complexes successfully catalyzed the Kumada reaction of aryl or heteroaryl diethyl phosphates with alkylmagnesium bromides (Scheme 9a) [59]. The reaction conditions in this study were predominantly used to prepare naphthalene decorated with alkyl substituents. Additionally, alkylated pyridine and 1,1’-biphenyl derivatives were also successfully prepared, as evidenced from selected examples **9**–**2b** and **9**–**2c**. In addition to the aforementioned protocol, Shen [60] and Sun [61] demonstrated that the cross-coupling reaction of organophosphates **9**–**3a** and **9**–**3b** with organobromides **9**–**4** can be conducted under reductive cross-coupling conditions (Scheme 9b).

**Scheme 10:**
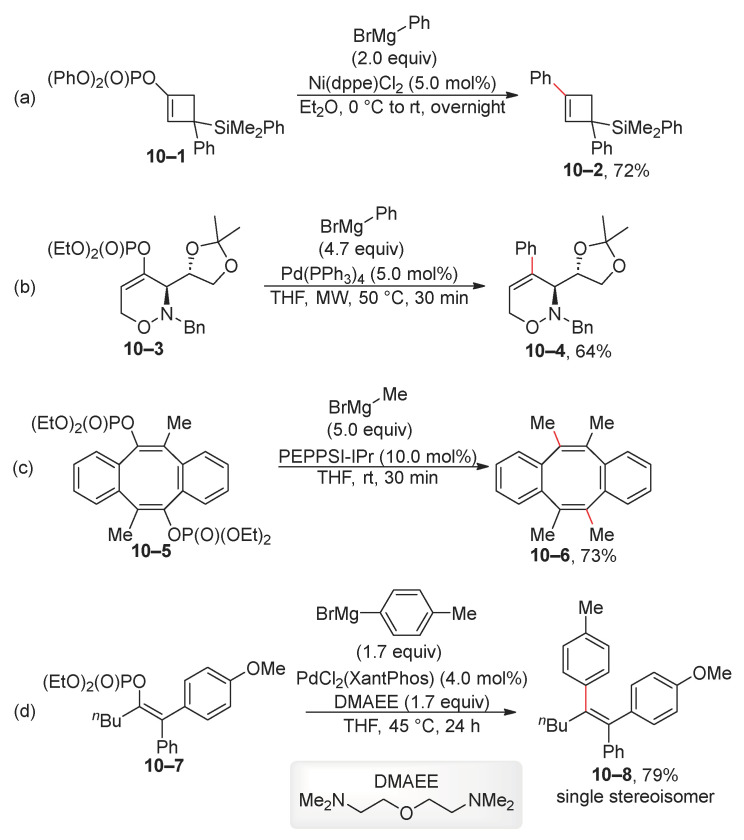
Palladium- and nickel-catalyzed Kumada reaction for the synthesis of (**a**–**c**) cyclic alkenes and (**d**) acyclic tetrasubstituted alkenes.

The Kumada reaction has also been used as a complementary method in some studies to broaden the scope of the research topic. Selected examples of this application are illustrated in Scheme 10. A substituted cyclobutene **10**–**2** was synthesized using a variation of the Mazet methodology (Scheme 10a) [62]. The Kumada reaction can be catalyzed via palladium complexes, as evidenced by the synthesis of cyclic alkenes **10**–**4** [63] and **10**–**6** [64] (Scheme 10b,c). Furthermore, Kotek published a stereoselective synthesis of tetrasubstituted alkenes from triply electrophilic templates, employing the Kumada reaction as the final step (Scheme 10d). Under optimized conditions, this method yielded tetrasubstituted alkene **10**–**8** as a single stereoisomer [65]. Brown [66] and Yoshikai [67] used similar conditions for the cross-coupling reaction of trisubstituted vinyl phosphates. In particular, the cross-coupling reaction of vinyl phosphates with Grignard reagents catalyzed by precatalysts, such as Ni(acac)_2_ [68], Fe(acac)_3_ [69,70], and Pd(PPh_3_)_4_ [71], has been used to synthesize a diverse range of alkenes.

**Scheme 11:**
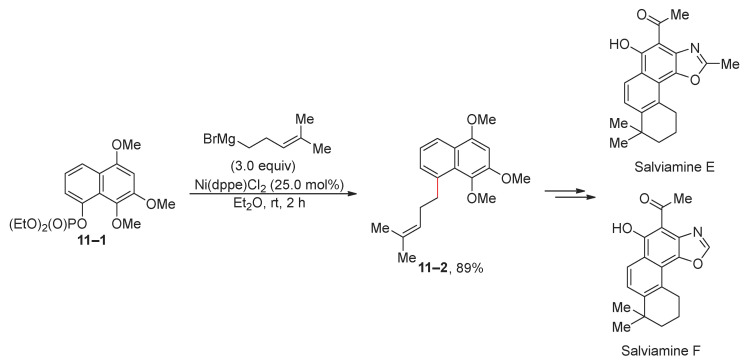
Synthesis of key intermediate during the total synthesis of salviamines E and F.

The Kumada reaction has been extensively used in the total syntheses of natural substances. An illustrative example is the nickel-catalyzed Kumada reaction, where naphthyl phosphate **11**–**1** reacts with an alkyl Grignard reagent (Scheme 11) [72]. Additionally, the Kumada reaction catalyzed through iron and nickel complexes was used to prepare a sex pheromone of Lobesia botrana [73], (–)-piperitylmagnolol [74], and indole alkaloids [75].

### 2.2. Negishi Reaction

**Scheme 12:**
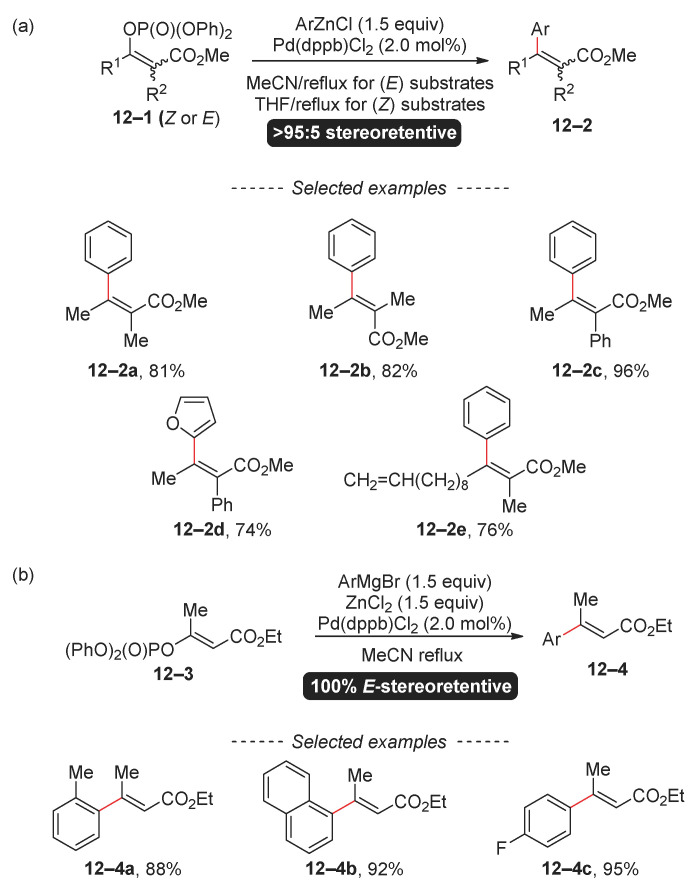
Application of the Negishi reaction for the synthesis of (**a**) tetrasubstituted and (**b**) trisubstituted alkenes.

The efficiency of the cross-coupling reaction involving organophosphates and organozinc, as well as trialkylaluminum reagents, is significantly influenced by the molecular structures of both organophosphate and organometallic reagents. For example, trialkyl aluminums can react with aryl diethyl phosphate at room temperature in the presence of a nickel complex [76]. In contrast, trisubstituted vinyl phosphates **12**–**1** undergo a reaction with organozinc reagents in refluxing THF or acetonitrile (Scheme 12) [77]. Under these conditions, the successful incorporation of heterocyclic and arylzinc halides, both with electron-donating and electron-withdrawing groups, was achieved. Similarly demanding conditions for the Negishi reaction of vinyl diphenyl phosphate **12**–**3** have been published by Shi and Xiao [78].

**Scheme 13:**
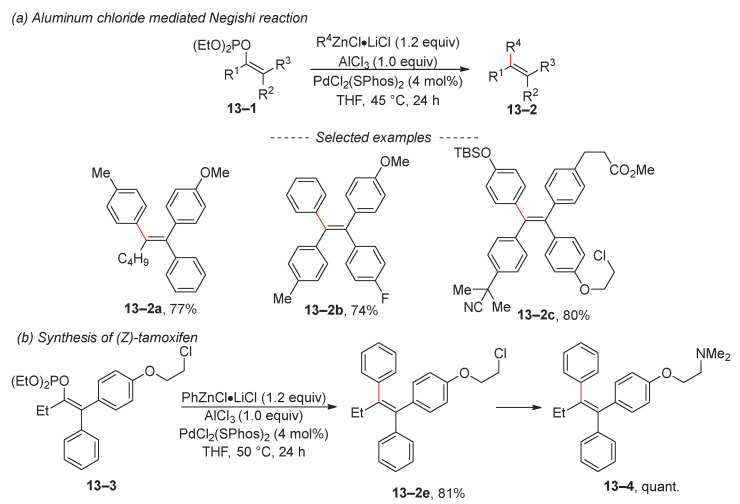
Aluminum chloride-mediated Negishi reaction of trisubstituted vinyl phosphate en route to (*Z*)-tamoxifen.

Recently, a novel approach for the stereoselective synthesis of tetrasubstituted alkenes was introduced (Scheme 13a) [79]. Starting phosphate **13**–**1** was synthesized via the double Suzuki coupling of dibromovinyl phosphates. Subsequently, the phosphate moiety was replaced via Negishi coupling in the presence of aluminum chloride. The developed reaction conditions tolerate various functional groups, including ester and nitrile groups. The authors proposed that the coordination of aluminum chloride with the phosphate group facilitates the oxidative addition of Pd(0) to the C–O bond. However, they noted that vinyl phosphate undergoes simultaneous decomposition in the presence of aluminum chloride. This methodology offers a straightforward and stereoselective route to (*Z*)-tamoxifen and its derivatives from easily available phosphate **13**–**3** (Scheme 13b).

**Scheme 14:**
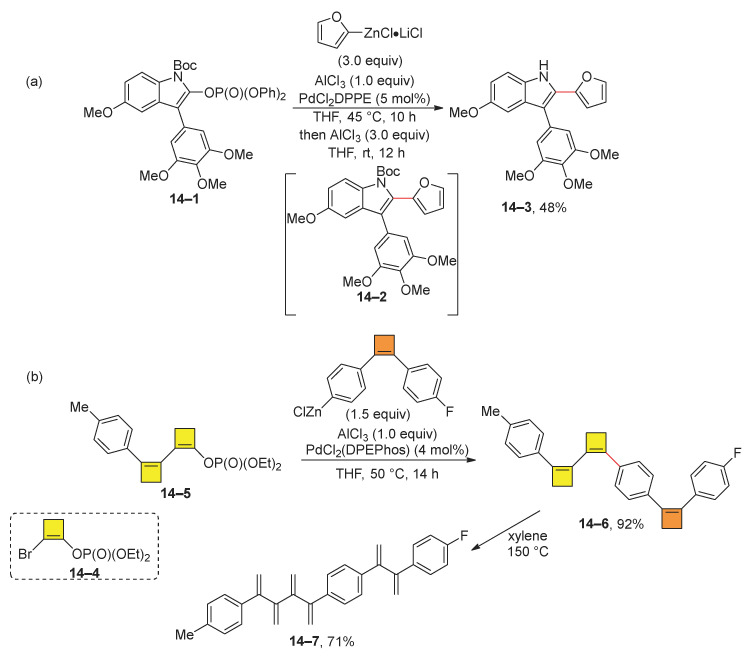
An aluminum chloride-promoted Negishi reaction for the synthesis of (**a**) substituted indole **14–3** and (**b**) [4]dendralene **14–7**.

Dibromovinyl or bromovinyl phosphates have been proven to be effective for the synthesis of substituted indoles and [n]dendralenes (Scheme 14). The synthesis of substituted indoles starts with Negishi coupling using **14**–**1**, which results in an indole derivative **14**–**2**. Subsequently, the synthesis of biologically active indole **14**–**3** was achieved by removing the Boc group using additional aluminum chloride (Scheme 14a) [80]. Furthermore, Scheme 14b illustrates the modular synthesis of [4]dendralenes by the Negishi reaction of cyclobutenyl phosphate **14**–**5** [81]. The starting cyclobutenyl phosphate **14**–**5** is synthesized from 2-bromocyclobutenyl phosphate **14**–**4**. Subsequent Negishi coupling yields cyclobutenyl derivatives **14**–**6**. An electrocyclic ring opening of this compound produced [4]dendralene **14**–**7**. A similar approach has been employed for the preparation of 2-substituted cyclobutanones [82] and cyclobutenones [83]. Further studies have shown that aluminum chloride facilitates the Negishi reaction of cycloalkenyl diphenyl phosphates at room temperature [84,85].

### 2.3. Suzuki Reaction

**Scheme 15:**
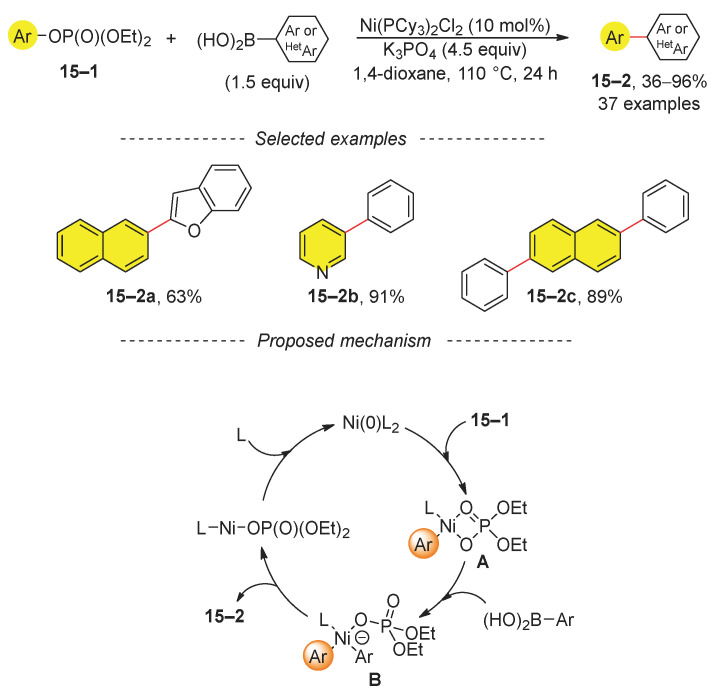
Nickel-catalyzed Suzuki reaction of aryl phosphates.

The Suzuki reaction has become a widely used tool for the formation of C–C bonds. The use of organophosphates in the Suzuki reaction has received significant attention in recent studies [86,87,88,89,90]. A recent example is the nickel-catalyzed transformation of aryl (diethyl) phosphates **14**–**1** in the presence of bis(tricyclohexylphosphine)nickel(II) dichloride (Scheme 15) [91]. This reaction is characterized by high yields of substituted biaryls. However, the scope of this reaction is limited to arylboronic acids. Vinyl and alkyl boronic acids were not used. The proposed mechanism for the published transformation involves the oxidative addition of a Ni(0)L_2_ complex, followed by transmetalation. The reaction product **15**–**2** is then produced by reductive elimination, and the nickel complex is regenerated by ligand substitution.

**Scheme 16:**
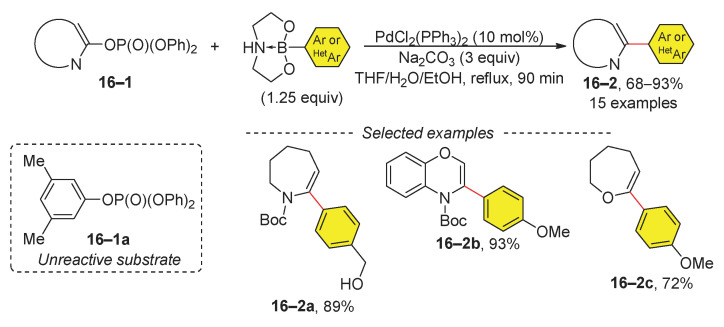
Palladium-catalyzed Suzuki reaction of phosphate enamides.

In contrast to diethyl phosphates, diphenyl phosphates are more common in the Suzuki reaction due to the higher reactivity of diphenyl phosphates. This is exemplified in a recent study presented in Scheme 16 [92]. Gigant and co-workers used dioxazaborocanes in a palladium-catalyzed reaction with phosphate enamides. The versatility of the reaction extends beyond the synthesis of azepine derivatives, since it accommodates a range of aromatic heterocyclic compounds, including substances **16**–**2b** and **16**–**2c**. However, the reaction conditions were specifically suited for vinyl phophates, and attempts to use aryl phosphates **16**–**1a** were unsuccessful. In addition, Senra and colleagues introduced an innovative palladium-supported layered double-hydroxide catalytic system for the Suzuki reaction of vinyl phosphates. Their research focused primarily on exploring the properties of this catalytic system; therefore, the scope of their reported reaction remains limited [93].

**Scheme 17:**
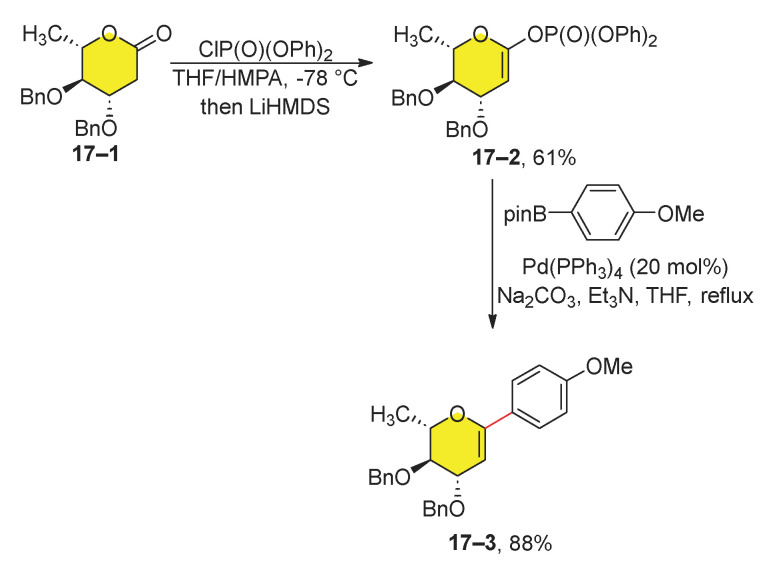
Suzuki reaction en route to *C*-arylglycal.

Recently, the Suzuki reaction between dihydropyranylphosphates **17**–**2** and 4-methoxyphenylboronic acid pinacol ester has been published (Scheme 17) [94]. Following careful optimization, it was determined that the most effective reaction conditions involved the use of Pd(PPh_3_)_4_ in THF under reflux. The optimized reaction conditions were used for the preparation of *C*-arylglycal **17**–**3**. The starting lactone **17**–**1** was synthesized from 4-di-*O*-acetyl-L-rhamnal and the phosphorylation itself was mediated by LiHMDS. However, the Suzuki reaction required 20 mol% of the catalyst to suppress the undesired chelation of the palladium atom to pyran oxygen. The final product **17**–**3** was obtained with a yield of 54% in two steps.

**Scheme 18:**
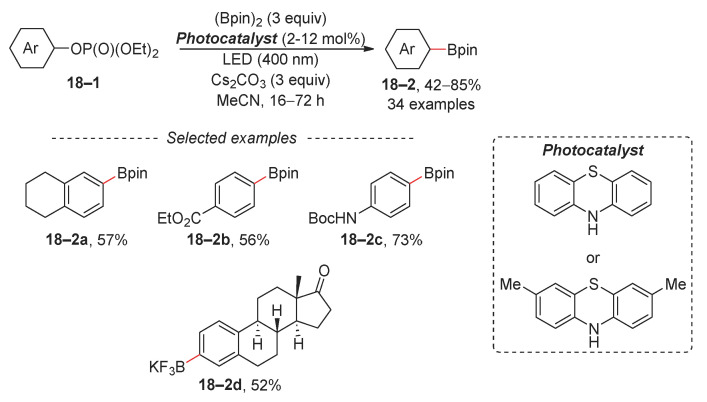
Visible light-mediated borylation of aryl and heteroaryl diethyl phosphates.

A popular variation of the Suzuki reaction is Miyaura borylation. This process typically involves the reaction of an electrophile with bis(pinacolato)diboron (Bpin-Bpin) in the presence of a catalytic amount of diverse transition-metal complexes [95,96]. Although organohalogens are the electrophiles of choice, organophosphates are also amenable to borylation. A recent example is the borylation of arylphosphates under photocatalytic conditions (Scheme 18) [97]. In this methodology, an aryl phosphate with a high negative reduction potential is activated via a phenothiazine photocatalyst, which exhibits enhanced reduction potential due to a proton-coupled electron transfer (PCET). Under optimized reaction conditions, a variety of aryl diethyl phosphates can be borylated, including esters and amides **18**–**2b** and **18**–**2c**. The synthesis of organotrifluoroborates is accessible; however, the crude reaction mixture after borylation requires treatment with KHF_2_ or KF. Furthermore, Li recently reported a metal-free borylation of aryl halides under continuous-flow conditions, although this method has a limited scope with respect to aryl diethylphosphates [98].

**Scheme 19:**
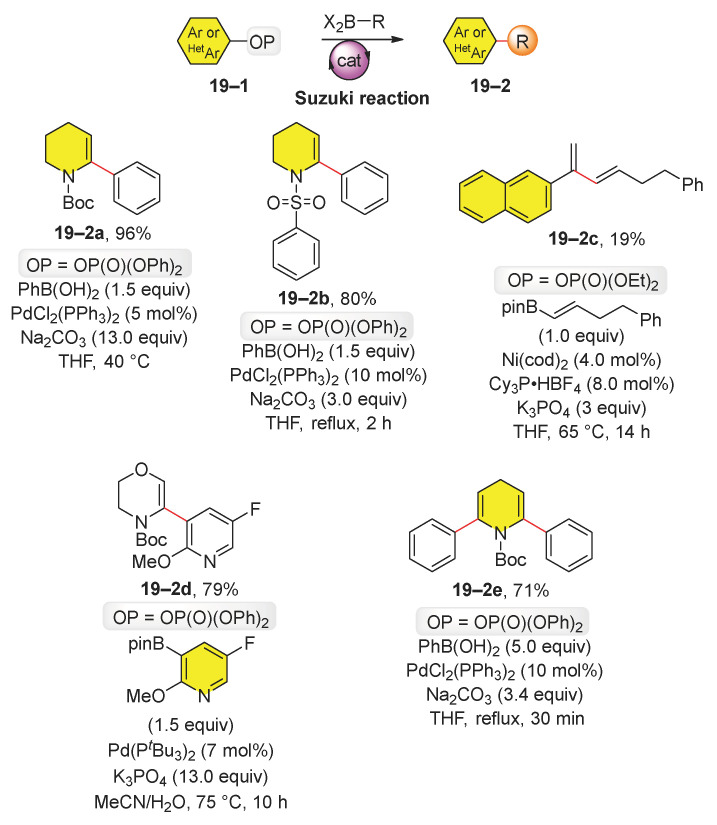
Application of the Suzuki reaction of organophosphates for the preparation of key intermediates.

The Suzuki reaction of organophosphates has been widely used for the preparation of various substances. Selected examples **19**–**2a** [99], **19**–**2b** [100], **19**–**2c** [101], and **19**–**2d** [102] are given in Scheme 19. In most cases, diphenyl phosphates were treated with aromatic and heteroaromatic boronic acids or their pinacol esters in the presence of a catalytic amount of palladium or nickel complexes. In addition, Asano and co-workers used the double Suzuki reaction for the synthesis of the heterocyclic compound **19**–**2e** [103].

**Scheme 20:**
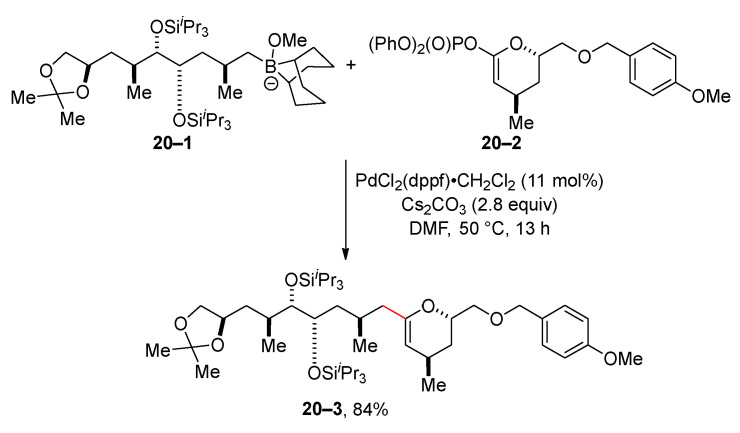
Suzuki coupling of cycloalkenyl phosphate en route to Didemnaketal B.

The application of the Suzuki reaction of organophosphates includes the synthesis of natural substances. Fuwa and Sasaki published a palladium-catalyzed Suzuki reaction of borane **20**–**1** with diphenyl phosphate **20**–**2** for the preparation of the dihydropyran derivative **20**–**3** en route to didemnaketal B (Scheme 20) [104]. The same authors used a similar Suzuki reaction to synthesize the C15–C38 fragment of akadaic acid [105].

**Scheme 21:**
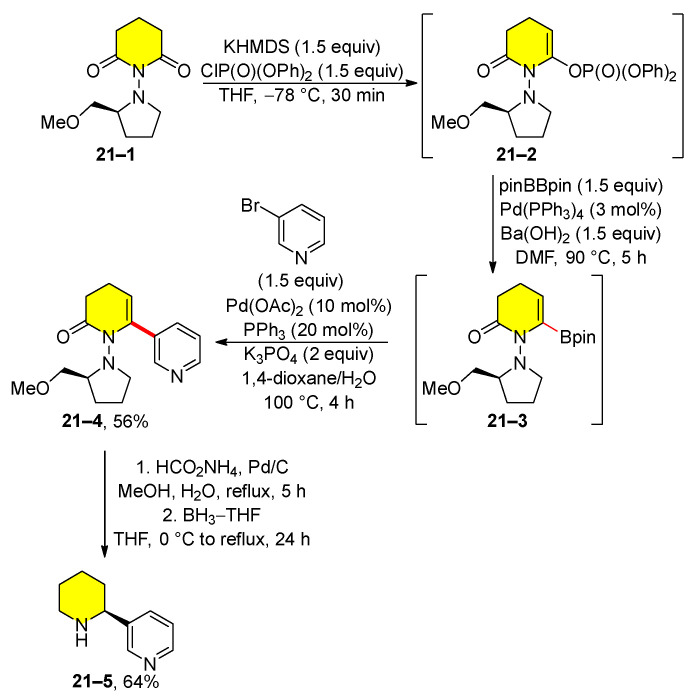
Total synthesis of (–)-anabasine.

In contrast, the published preparation of (–)-anabasine (**21**–**5**) took advantage of the palladium-catalyzed Suzuki–Miyaura borylation. This process was applied to crudely isolated phosphate **21**–**2** in the presence of barium hydroxide (Scheme 21) [106]. The prepared pinacol boronic acid ester **21**–**3** was treated with 3-bromopyridine to give alkene **21**–**4** with a 56% isolated yield. Finally, (–)-anabasine was obtained by reducing cycloalkene **21**–**4**.

### 2.4. C–H Activation

**Scheme 22:**
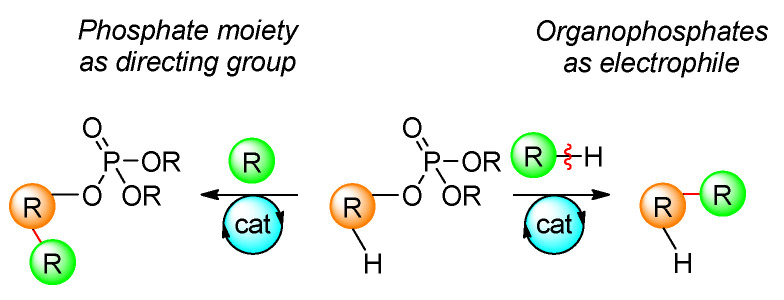
General scheme representing the use of organophosphates for the activation of the C–H bond.

Organophosphates are also widely used in the transition-metal-catalyzed activation of the C–H bond. The phosphate group can serve as a directing group to facilitate the activation of the C–H bond. Alternatively, organophosphates are used as electrophiles for the C–H bond activation (Scheme 22).

**Scheme 23:**
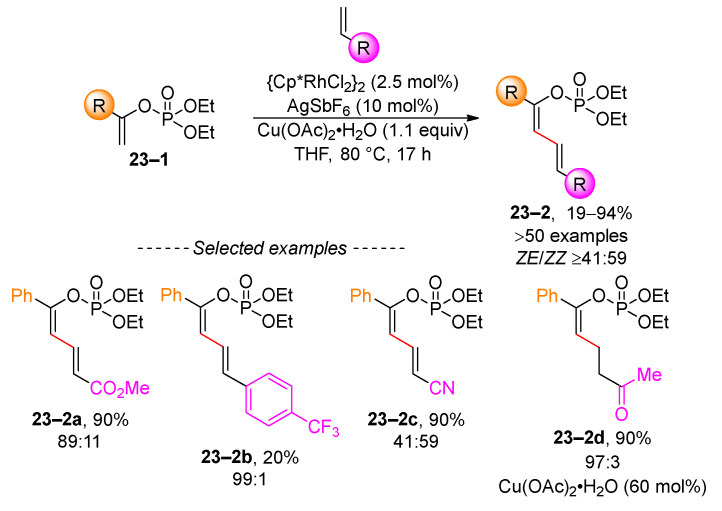
Rhodium-catalyzed vinylation of vinyl phosphates.

An example of C–H-bond activation involves the rhodium-catalyzed vinylation of vinyl phosphates **23**–**2** (Scheme 23) [107]. A comprehensive series of dienyl diethyl phosphates was prepared under optimized reaction conditions. Although the scope of the reaction was extensively explored with methyl acrylate, styrene derivatives also performed similarly, as illustrated in example **23**–**2b**. However, the reaction with acrylonitrile resulted in the vinylation product **23**–**2c,** albeit with a reduced *ZE*/*ZZ* ratio. Adjusting the amount of copper acetate to 60 mol% and the use of enones led to the formation of hydroalkylation product **23**–**2d**.

**Scheme 24:**
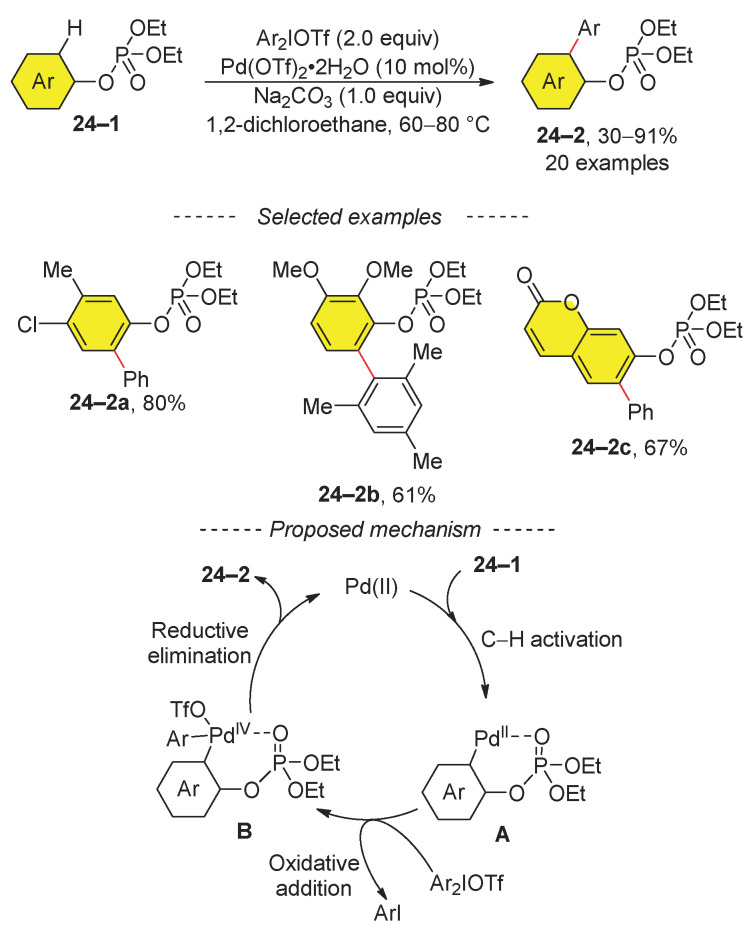
Palladium-catalyzed arylation of aryl phosphates via iodonium salts.

The phosphate-directing group has been applied to the synthesis of biaryls **24**–**2** from aryl diethyl phosphates **24**–**1** (Scheme 24) [108]. However, the scope of this reaction is limited to the preparation of simple biaryls and cyclic lactone **24**–**2c**. The proposed mechanism involves the formation of Pd(IV) complex **B**, which then undergoes reductive elimination to yield the coupling product **24**–**2**.

**Scheme 25:**
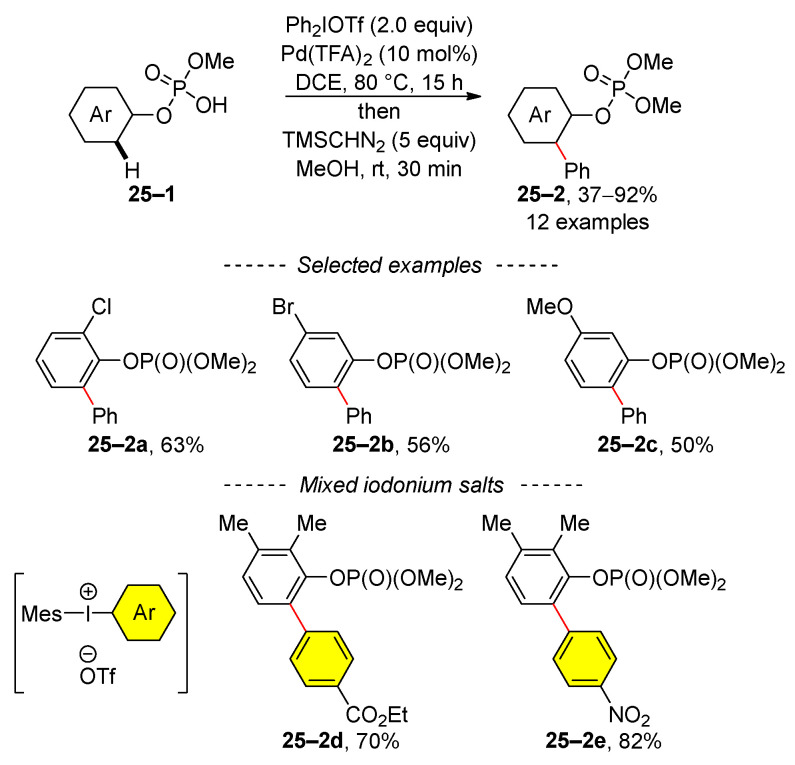
Palladium-catalyzed C–H bond activation directed via monophosphoric acid group.

In addition to the dialkyl phosphate group, a monophosphoric acid-directing group can also facilitate the activation of the C–H bond (Scheme 25) [109]. The reaction scope was assessed using diphenyliodonium triflate in the presence of a catalytic amount of palladium trifluoroacetate. However, arylation with mixed iodonium salts is possible, as illustrated by the selected examples **25**–**2d** and **25**–**2e**. The authors assumed that the C–H-bond-activation reaction involved the Pd(II)↔Pd(IV) catalytic cycle.

**Scheme 26:**
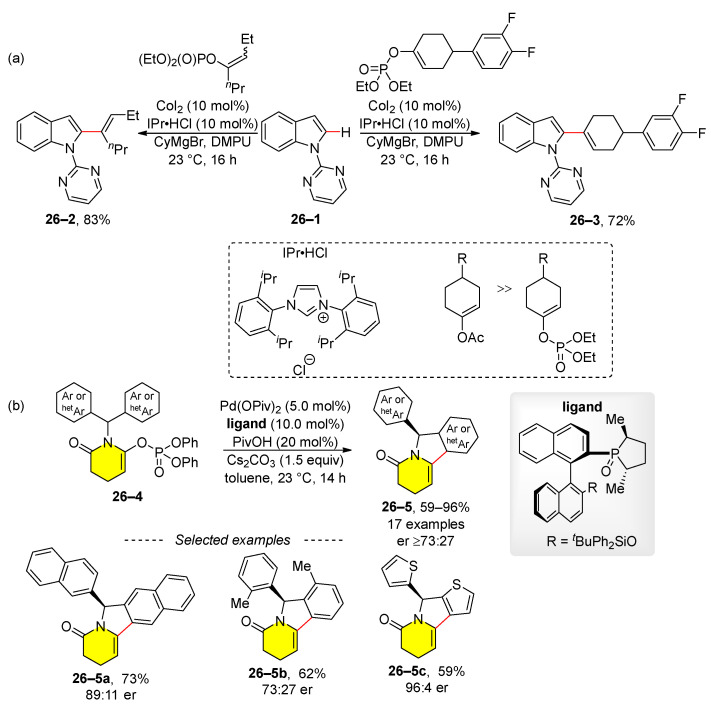
Vinyl phosphates as electrophiles in (**a**) cobalt-catalyzed C−H-activation of substituted indole and (**b**) intramolecular cyclization of vinyl phosphate.

Organophosphates are also used as electrophiles in C–H-bond activation. In a series of studies, Ackermann and co-workers investigated vinyl acetates, phosphates, and carbonates as electrophiles for the cobalt-catalyzed C–H bond activation of indoles (Scheme 26a) [110,111]. The optimized reaction conditions included the use of an *N*-heterocyclic carbene (NHC) ligand, enabling the introduction of an alkenyl group from vinyl acetates at room temperature. Diethyl vinyl phosphates are effective electrophiles, as demonstrated by examples **26**–**2** and **26**–**3**. Unfortunately, the scope of the reaction is limited to simple vinyl phosphates despite the availability of a wide range of functionalized vinyl phosphates. It is worth noting that cyclohexyl acetates exhibit higher reactivity compared to their cyclohexenyl phosphate counterparts. Besides cobalt-catalyzed C–H-bond activation, Cramer reported the enantioselective palladium-catalyzed cyclization of ketene aminal phosphate, leading to an indolizidine scaffold (Scheme 26b) [112].

**Scheme 27:**
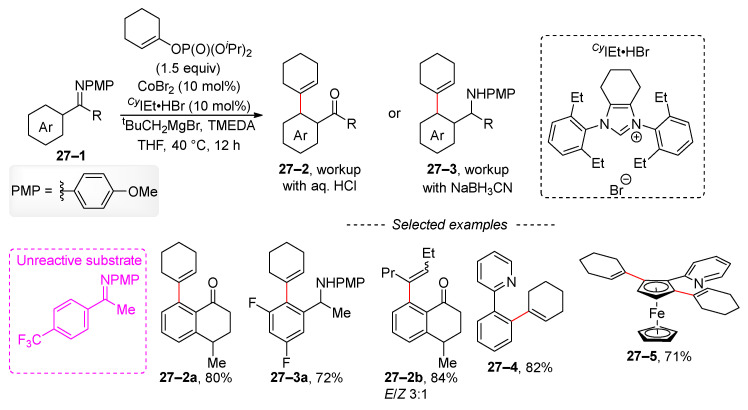
Cobalt-catalyzed vinylation of benzylic imines.

Another cobalt-catalyzed C–H-bond activation involves aromatic imines **27**–**1** (Scheme 27) [113]. Similar to Ackermann’s work, the NHC ligand, whose structure is shown in Scheme 26, is also used in this case. The advantage of the imino-directing group lies in its ability for late-stage transformation, either by hydrolysis into ketone **27**–**2** or reduction to a secondary amine **27**–**3**. Cyclohexenyl phosphates were used predominantly because the formation of a mixture of *E* and *Z* isomers was observed for the acyclic derivative **27**–**2b.** Furthermore, an imine with an electron-withdrawing trifluoromethyl group was unreactive. The optimized reaction conditions were effectively applied to substrates with a 2-pyridyl directing group, as exemplified by the synthesis of compounds **27**–**4** and **27**–**5**. In a subsequent publication, the same group applied cobalt-catalyzed C–H-bond activation to unprotected imines [114]. In this publication, a mechanism for the studied reaction is also presented.

**Scheme 28:**
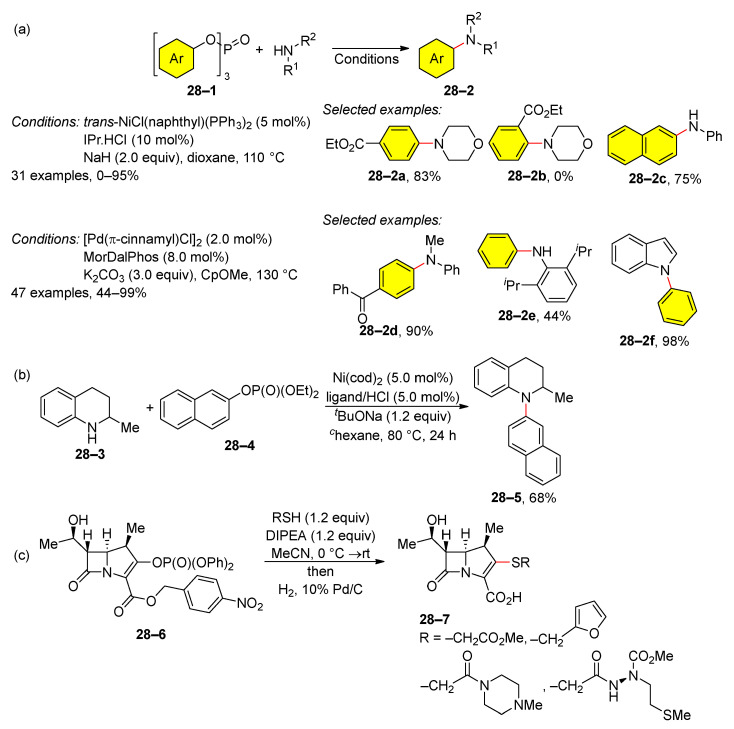
Nickel- and palladium-catalyzed synthesis of (**a**) of amines, (**b**) substituted 2-naphthylamine **28–5**. Transition-metal-free synthesis of carbapenems (**c**).

In addition to previous transition-metal-catalyzed reactions of organophosphates, the concept of reactions catalyzed by transition-metal complexes was also used for the preparation of aromatic amines from triaryl phosphates (Scheme 28a). The nickel-catalyzed arylation of amines proceeds in dioxane at 110 °C [115]. The optimized reaction conditions are characterized by a wide reaction scope; however, the conditions failed to deliver a substrate with an ethoxycarbonyl group in the *ortho* position **28**–**2b**. The palladium-catalyzed amination of organophosphates encompasses a broad spectrum of N-heterocyclic compounds, such as indole derivatives marked as **28**–**2f** [116]. In addition, the same catalytic conditions include [Pd(2-butenyl)Cl]_2_ along with the MorDalPhos ligand to facilitate the α-arylation of aryl and heteroaryl ketones [117]. Recently, the nickel-catalyzed amination of organohalides was reported by Hong and Shi. The optimized reaction conditions are suitable for the amination of carbonate, tosylate, carbamate and organophosphate **28**–**4** (Scheme 28b) [118]. In addition, the synthesis of carbapenems **28**–**7** was realized by the substitution of the diphenyl phosphate group for the corresponding thiol (Scheme 28c) [119].

## 3. Organophosphate Rearrangements

**Scheme 29:**
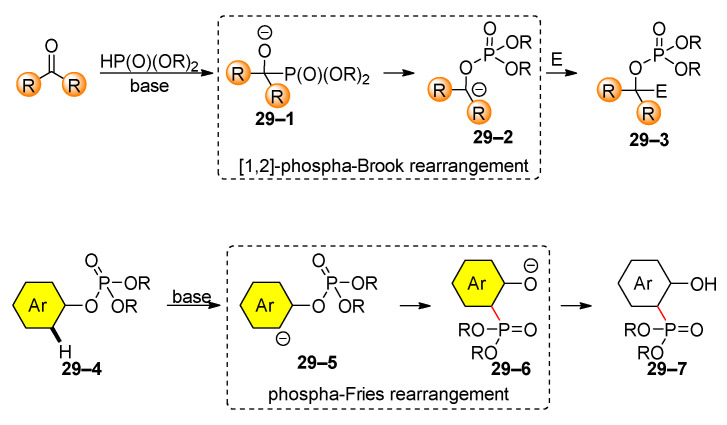
Schematic representation of the [1,2]-phospha-Brook and phospha-Fries rearrangements.

Organophosphates are involved in two types of rearrangements. The first rearrangement is the [1,2]-phospha-Brook rearrangement, which follows the traditional Brook rearrangement [120,121,122]. This process starts with the reaction of a carbonyl compound with dialkylphosphite in the presence of a base (Scheme 29). The initially formed phosphonate **29**–**1** undergoes rearrangement to produce an alkyl phosphate **29**–**2**, which is then amenable to a further reaction with an electrophile. Terada’s group has extensively used the [1,2]-phospha-Brook rearrangement for the synthesis of substituted furans [123,124], pyrroles [125], phenantherenes [126], indolizine [127], piperidines [128], oxindoles [129], chromenes [130], and many others [131,132,133,134,135,136,137]. The [1,2]-Phospha-Brook rearrangement has also attracted significant attention from other research groups, leading to numerous studies [138,139,140,141,142] and comprehensive reviews [143,144]. The second rearrangement associated with organophosphates is the anionic phospha-Fries rearrangement. First reported by Melvin in 1981 [145], this rearrangement typically involves the isomerization of aryl phosphates to aryl phosphonates in the presence of a base, typically LDA. Since its discovery, the anionic phospha-Fries rearrangement has been used as a cornerstone to synthesize lithium borates as promising materials for lithium-ion batteries [146] and receptors for the complexation of lanthanides [147] and other transition metals [148,149]. Recently, Snieckus’s group reported an anionic phospha Fries rearrangement of aryl tetraethylphosphorodiamidates [150,151]. A review of the phospha-Fries rearrangement was published in 2005 [152]; therefore, this chapter summarizes recent findings in this area.

**Scheme 30:**
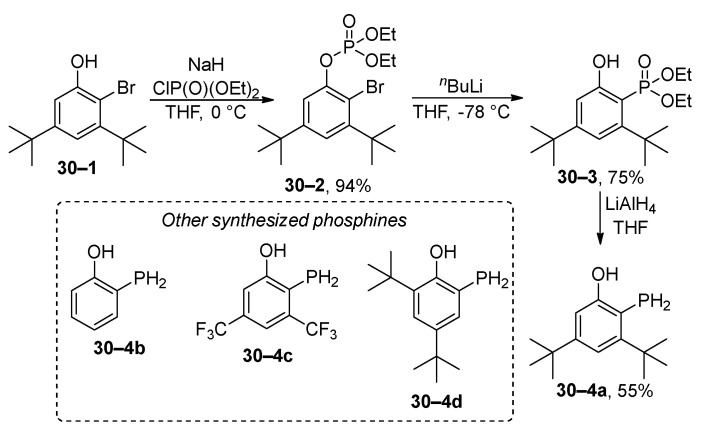
Synthesis of monoaryl phosphines by anionic phospha-Fries rearrangement.

The phospha-Fries rearrangement has been used for the preparation of monoarylphosphines (Scheme 30) [153]. Initially, the starting ortho-bromophenol **30**–**1** is converted to phosphate **30**–**2**. Subsequently, the phospha-Fries rearrangement is initiated by a bromine–lithium exchange reaction. The formed phosphonate **30**–**3** is reduced, using lithium aluminum hydride to phosphine **30**–**4a** in a total isolated yield of 39% (three steps). In addition to the model example **30**–**4a**, three additional monoaryl phosphines **30**–**4b**, **30**–**4c,** and **30**–**4d** were prepared. However, the prepared phosphines have limited stability and decompose to phenol and phosphine. The authors proposed that the decomposition of phosphines **30**–**4** is catalyzed by traces of acid originating from the workup of the reduction of phosphonates **30**–**3**.

**Scheme 31:**
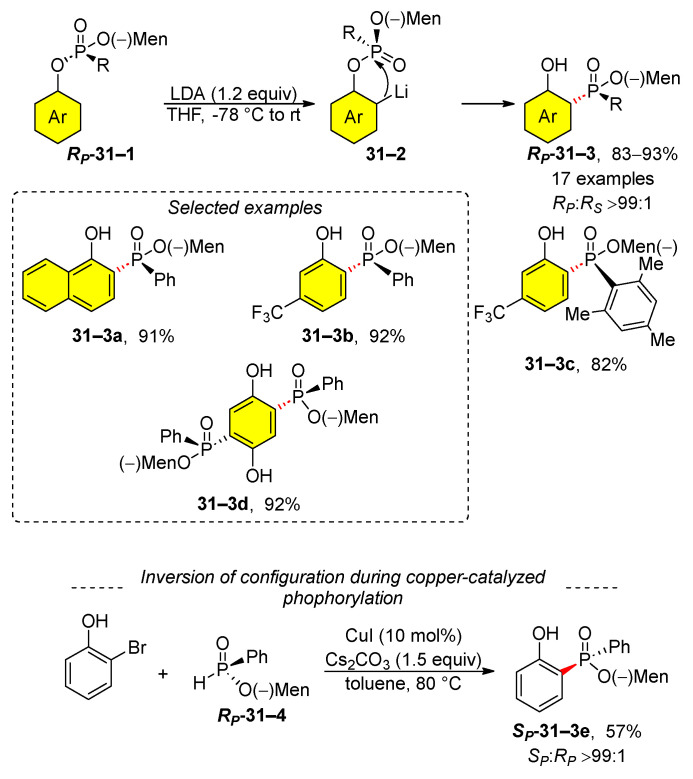
Stereoretentive phospha-Fries rearrangement.

In 2014, Han and Yin published a study on a copper-catalyzed phosphorylation of 2-halophenols en route to substituted phenols [154]. As part of their research, they studied the phospha-Fries rearrangement of aryl phosphinates that contain (–)-menthol **31**–**1** (Scheme 31). An important aspect of their findings is that the reaction proceeded with complete retention of configuration at the phosphorus atom. Thus, the phosphate ***R_P_*-31**–**1** phosphinate is transformed into phosphonate ***R_P_*-31**–**3**. In contrast, they also showed that the cross-coupling of 2-bromophenol with phosphinate ***R_P_*-31**–**4** gave the phosphonate ***S_P_*-31**–**3e** in a moderately isolated yield.

**Scheme 32:**
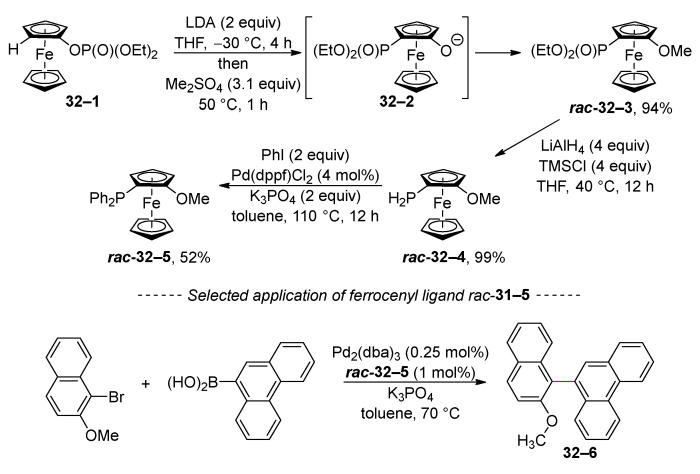
Synthesis of ferrocenyl ligand ***rac*-32**–**5** and its application in the Suzuki cross-coupling reaction.

The anionic phospha-Fries rearrangement was also used to prepare the ferrocene ligand ***rac*-32**–**5** (Scheme 32) [155]. The starting phosphate **32**–**1** is isomerized to the anion **32**–**2**. Subsequent alkylation with dimethyl sulphate yields ferrocenyl ether ***rac*-32**–**3**. The phosphonate group of ferrocene ***rac*-32**–**3** is then reduced with lithium aluminum hydride. Subsequent arylation through Stelzer P,C cross-coupling [156] gave the desired phosphine ***rac*-32**–**5**. The isolated phosphine ***rac*-32**–**4** has been used successfully in Suzuki coupling to synthesize the corresponding biaryls. This application is exemplified by the representative synthesis of biaryl **32**–**6**, which involves the reaction of phenanthren-9-ylboronic acid with 2-methoxy-1-bromonaphthalene.

**Scheme 33:**
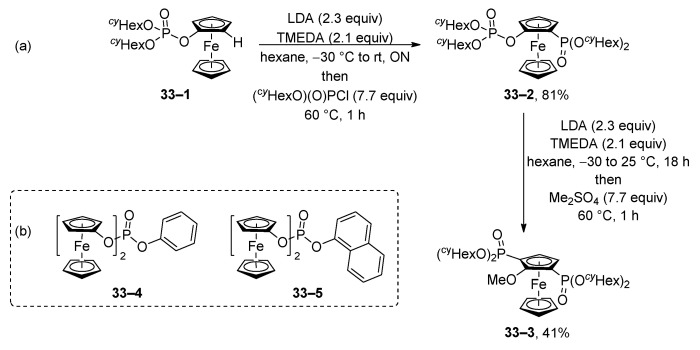
Phospha-Fries rearrangement of (**a**) symmetrical ferrocenyl bisphosphates and (**b**) unsymmetrical bisphosphates.

The same group studied the phospha-Fries rearrangement of ferrocenyl bisphosphates [157]. In this study, they found that bisphosphonates can be synthesized by sequential phospha-Fries rearrangement (Scheme 33). Initially, ferrocenyl phosphate **33**–**1** undergoes rearrangement to form phosphonophosphate **33**–**2**. This intermediate is then subjected to a second phospha-Fries rearrangement to give the final product **33**–**3**. The behavior of mixed phosphates **33**–**4** and **33**–**5** was also investigated. However, the rearrangement of phosphates **33**–**4** and **33**–**5** resulted in a product mixture demonstrating limited chemoselectivity [158].

**Scheme 34:**
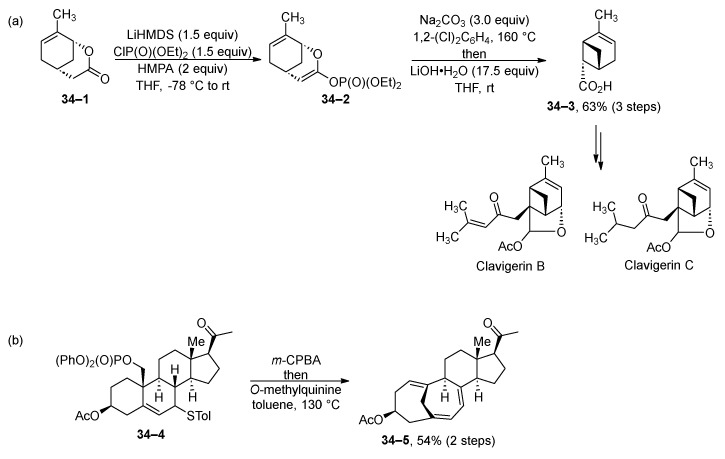
(**a**) Ireland-Claisen rearrangement of vinyl phosphate en route to clavigerins B and C. (**b**) Base-mediated phosphate elimination in the synthesis of cyclocitrinols.

The rearrangement of organophosphates has found applications in the total synthesis of natural products. A notable example is the Ireland–Claisen rearrangement of vinyl phosphate **34**–**2** used in the synthesis of clavigerins B and C (Scheme 34a) [159]. After rearrangement, the hydrolysis of the crude reaction mixture yielded carboxylic acid **34**–**3** with a 63% yield. Furthermore, the preparation of cyclocitrinols makes use of an optimized version of alkyl diphenyl phosphate rearrangement (Scheme 33b) [160,161]. This rearrangement begins with the oxidation of sulfide **34**–**4** to the corresponding sulfoxide. Then, the sulfoxide was rearranged to intermediate **34**–**5**.

**Scheme 35:**
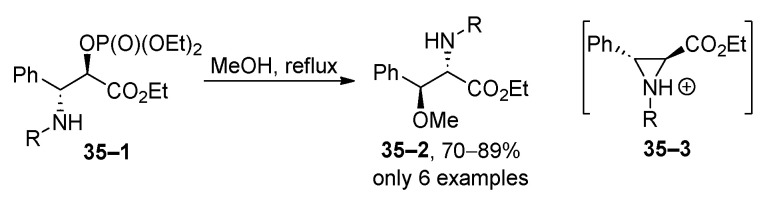
Rearrangement of aliphatic diethyl phosphate **34**–**1** to α-amino acid.

Kaabi and Besbes documented the rearrangement of aliphatic diethyl phosphates **35**–**1** into α-amino acids **35**–**2** (Scheme 35) [162]. While the scope of the reaction is confined primarily to secondary amines with simple alkyl substituents, the authors propose that the formation of an aziridinium ion **35**–**3** plays a crucial role as an intermediate in this transformation.

## 4. Transition-Metal-Free Substitution Reactions of Organophosphates

**Scheme 36:**
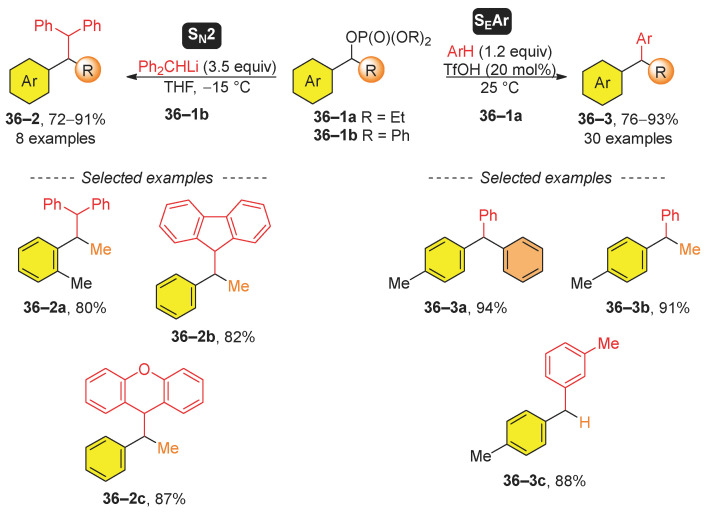
Benzyl phosphates as precursors for the preparation of aromatic hydrocarbons.

Recent studies have introduced two distinct methods for the substitution of benzyl phosphates (Scheme 36). In the first method [163], diphenylmethyllithium was used as a nucleophile for the S_N_2 substitution of diphenyl phosphates **36**–**1a**. Moreover, the authors also prepared two advanced aromatic hydrocarbons, **36**–**2b** and **36**–**2c**. The S_N_2 nucleophilic substitution mechanism was confirmed using an enantiomerically pure benzyl phosphate, which underwent substitution proceeded with inversion of configuration. The same group further broadened the scope of the reaction to cycloalkenyl and propargyl phosphates [164]. In contrast, Chakravarty [165] reported the Friedel-Crafts alkylation of aromatic hydrocarbons [165]. The authors used a catalytic amount of trifluoromethanesulfonic acid (TfOH) to generate a benzyl carbocation that subsequently reacted with an aromatic hydrocarbon (ArH). The efficacy of this reaction is demonstrated through the synthesis of selected products **36**–**3a**, **36**–**3b**, and **36**–**3c**. Following previous work [166], Yamamoto’s group developed a ferric triflate-catalyzed method for the formation of (difluoromethyl)(diaryl)methanes from the corresponding 2,2-difluoro-1-arylethyl phosphates [167].

**Scheme 37:**
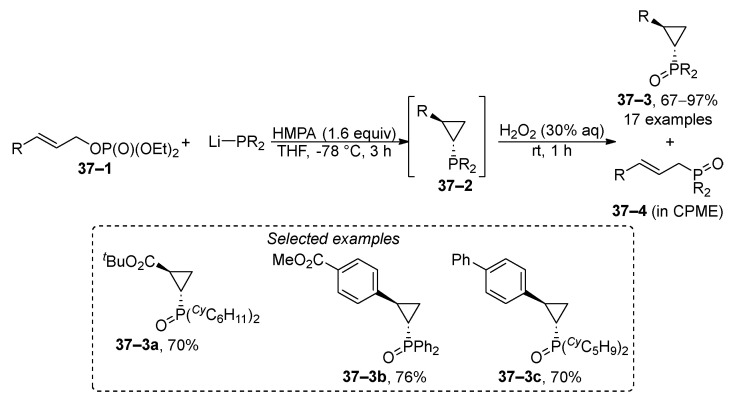
Synthesis of cyclopropylphosphines by the cyclopropanation of allyl phosphates.

In 2020, a novel cyclopropanation reaction was introduced for the preparation of cyclopropylphosphinoxides (Scheme 37) [168]. The formation of cyclopropane derivatives is achieved by the interaction of allyl diethyl phosphates **37**–**1** with lithium phosphides in tetrahydrofuran. Upon the completion of the reaction, the resulting phosphines **37**–**2** are oxidized to stable phosphinoxides **37**–**3**. An interesting aspect of this reaction is its sensitivity to the choice of solvent. For example, the use of cyclopentyl methyl ether (CPME) results in the formation of an S_N_2 nucleophilic substitution product **37**–**4**, whereas tetrahydrofuran preferentially yields the cyclopropanation product **37**–**3**. Further experiments indicate that the selectivity between the cyclopropanation product **37**–**3** and S_N_2 product **37**–**4** is influenced by the electrophilicity at the β-position of the allylic moiety.

**Scheme 38:**
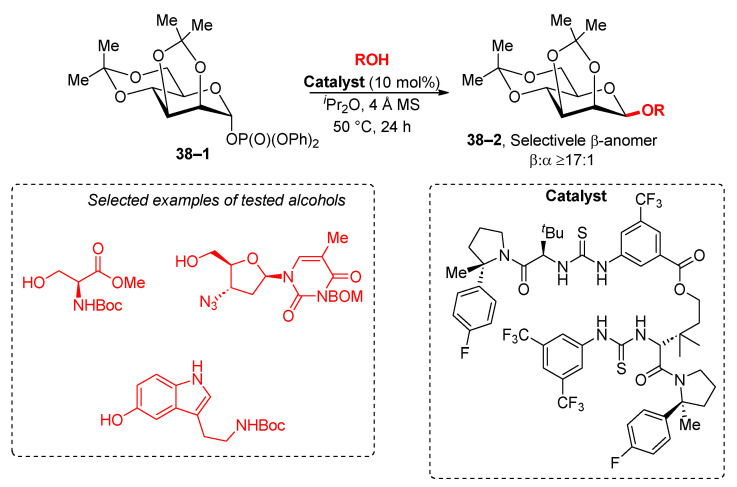
β-Selective glycosylation of glycosyl phosphates through dual activation mode.

Scheme 38 illustrates the application of the catalytic activation of the phosphate group for β-selective glycosylation [169,170]. Central to this process is a bifunctional and chiral catalyst. This catalyst serves a dual role: it activates the phosphate group via coordination and facilitates in the proton abstraction from the alcohol. When the phosphate group is activated by Lewis acid, followed by a reaction with a corresponding alcohol, the product is predominantly in the thermodynamically more stable α-anomer form. Selected examples of tested alcohols show that phenols can also be used.

**Scheme 39:**
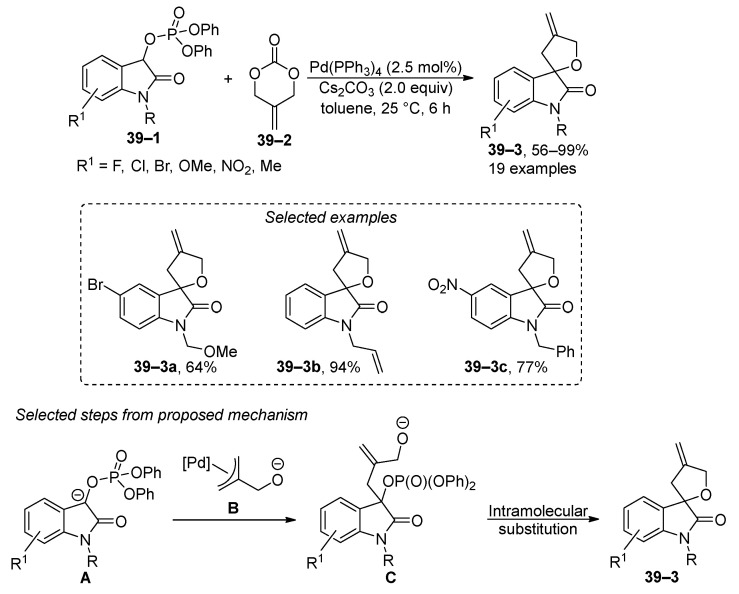
[4 + 1] cyclization of 2-oxindolyl phophates en route to spirooxindolin derivatives.

Intramolecular substitution at the phosphate group at 2-oxoindolinyl phosphates **39**–**1** has been used to synthesize spiroindolines **39**–**3** (Scheme 39) [171]. In this study, the authors proposed that the reaction starts with the activation of carbonate **39**–**2** through the formation of allyl complex **B**. Complex **B** then reacts with deprotonated 2-oxindoline **A** to form the expected intermediate **C.** Subsequent intramolecular substitution at the phosphate moiety results in the final product **39**–**3**. However, it is important to note that the reaction’s scope is limited to a few functional groups, which are enumerated in Scheme 39. An exploratory experiment focusing on the chiral induction of spirooxindoline yielded moderate enantioselectivity. A comparable methodology has been used for the synthesis of pyrano [2,3-b]indol-2-ones, with the oxindoline phosphates being generated in situ [172].

**Scheme 40:**
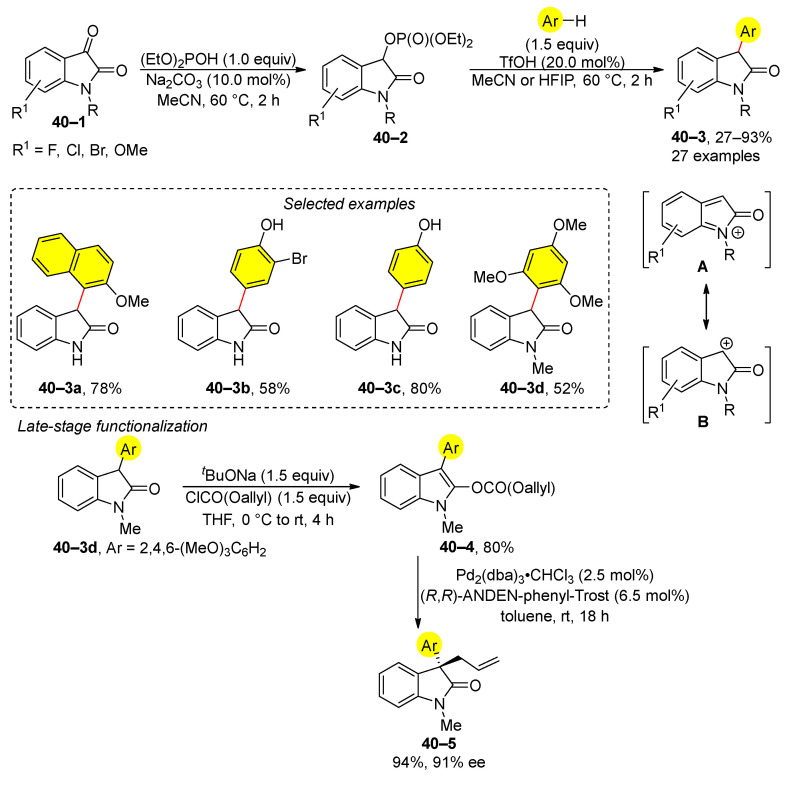
One-pot two-step synthesis of aryl 2-oxindoles from organophosphates.

The conversion of isatin into 2-oxindol-3-yl phosphates facilitates the synthesis of 3-aryl-2-oxindoles, as illustrated in Scheme 40 [173]. The starting phosphate **40**–**2** was synthesized via a base-catalyzed phospha-Brook rearrangement. This is followed by the preparation of 3-aryl-2-oxindole **40**–**3** by Friedel-Crafts alkylation, initiated via a catalytic amount of trifluoromethanesulfonic acid. An important intermediate during the conversion of **40**–**2** to **40**–**3** is cation **A** (due to the delocalization of the cation). The reaction can be carried out in either acetonitrile or hexafluoropropan-2-ol (HFIP), but higher yields are typically achieved in HFIP. Due to the specific requirements of the Friedel-Crafts alkylation, only electron-rich aromatic compounds are suitable for this reaction, as illustrated by selected examples. The synthesized 3-aryl-2-oxindole **40**–**3** offers the potential for further transformations. For example, 2-oxindole **40**–**3 d** was converted into the corresponding carbonate **40**–**4** by nucleophilic substitution. This was followed by a palladium-catalyzed asymmetric intramolecular allylic alkylation in the presence of (*R*,*R*)-ANDEN-phenyl-Trost ligand to produce 2-oxindole derivative **40**–**5** with a chiral quaternary center.

**Scheme 41:**
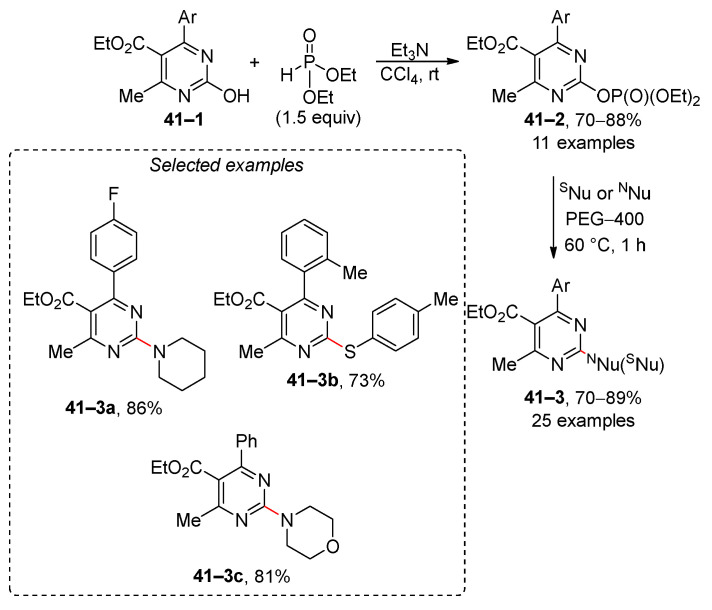
Nucleophilic aromatic substitution of pyrimidyl phosphate.

It has been demonstrated that heteroaromatic diethyl phosphates are effective in nucleophilic substitution reactions (Scheme 41) [174]. The starting compound **41**–**1** was converted to phosphate **41**–**2** by the reaction of 2-hydroxypyrimidines with diethyl phosphonate. The resulting phosphate **41**–**2** subsequently undergoes a reaction with dialkylamines or 4-tolylthiol, yielding the trisubstituted pyrimidine **41**–**3** in high yields. However, the scope of this reaction is limited to pyrimidine derivative **41**–**2**.

## 5. Transition-Metal-Catalyzed Allylic and Benzylic Substitution of Organophosphates

**Scheme 42:**
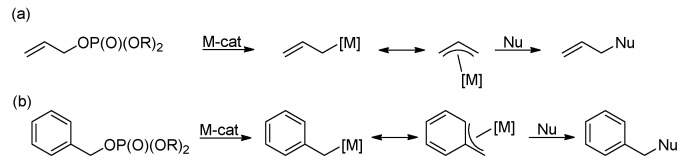
Transition-metal-catalyzed allylic substitution of (**a**) allyl and (**b**) benzyl phosphates.

The method of the transition-metal-catalyzed substitution of organophosphates typically involves the reaction of allyl or benzyl phosphates with a suitable nucleophile in the presence of a catalytic amount of a transition-metal complex (Scheme 42a,b). Since transition-metal-catalyzed allyl substitution has been the subject of several reviews [175,176,177,178,179], the aim of this section is to concisely summarize the fundamental applications of allyl and benzyl phosphates in the realm of transition-metal-catalyzed allylic substitution.

**Scheme 43:**
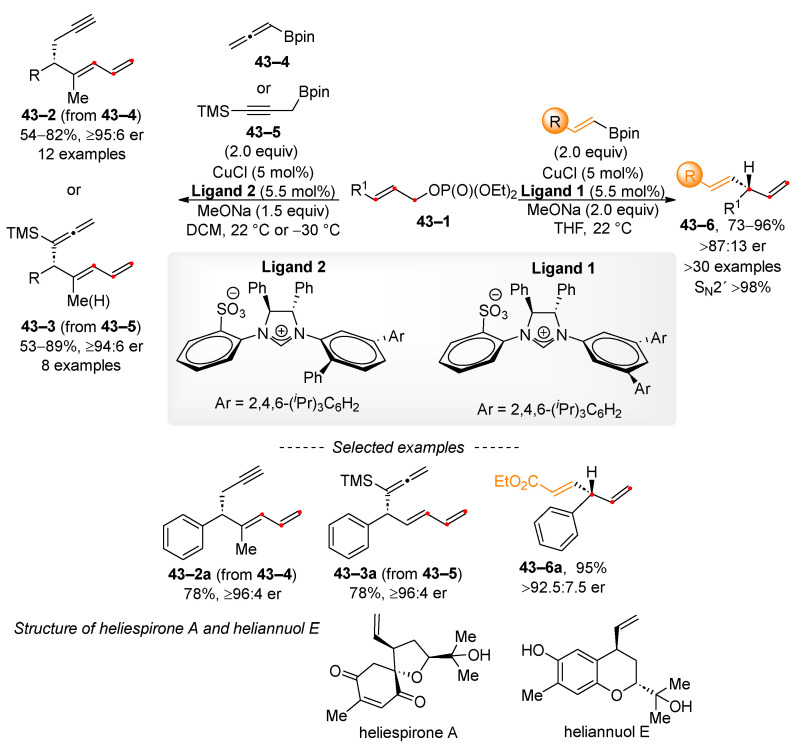
Copper-catalyzed reaction of allyl diethyl phosphates with boronic acid pinacol esters.

A recent example of transition-metal-catalyzed allylic substitution of allyl phosphates is the asymmetric copper(I)-catalyzed substitution between allyl diethyl phosphates **43**–**1** and alkenyl boronates (Scheme 43) [180]. In this reaction, the starting phosphate **43**–**1** is efficiently transformed into skipped dienes **43**–**6** in high yields and excellent enantioselectivity. The authors further demonstrated the versatility of this methodology by applying it to heterocycloalkenyl boronates. The product structure **43**–**6** aligns with an S_N_2′ nucleophilic substitution that is the predominant reaction pathway. Optimized reaction conditions were used for the formal synthesis of natural substances, such as heliespirone A and heliannuol E. The same group highlighted the unique effectiveness of **ligand 1** in the enantioselective S_N_2′ substitution of allyl diethyl phosphate **43**–**1** with cuprated ethylboronic acid pinacol ester. This ester is synthesized via the in situ addition of an L_n_Cu-H complex to a vinylboronic acid pinacol ester [181]. The use of allenyl boronate **43**–**4** and propargyl boronate **43**–**5** in the same reaction was explored by Hoveyda (Scheme 43) [182]. The dienyl phosphate **43**–**1** is converted into the corresponding products **43**–**2** and **43**–**3,** with the outcome depending on the structure of the boronate. Once again, the reaction predominantly follows an S_N_2′ pathway with high enantioselectivity, which is influenced by the chosen ligand. The observed reactivity of allyl phosphate **43**–**1** with boronates **43**–**4** and **43**–**5** contrasts with a previous study [183]. In this study, an NHC ligand combined with copper(II) chloride was used to transform the phosphate **43**–**1** and alkyne **43**–**5** into disubstituted acetylene following S_N_2′ regioselectivity.

**Scheme 44:**
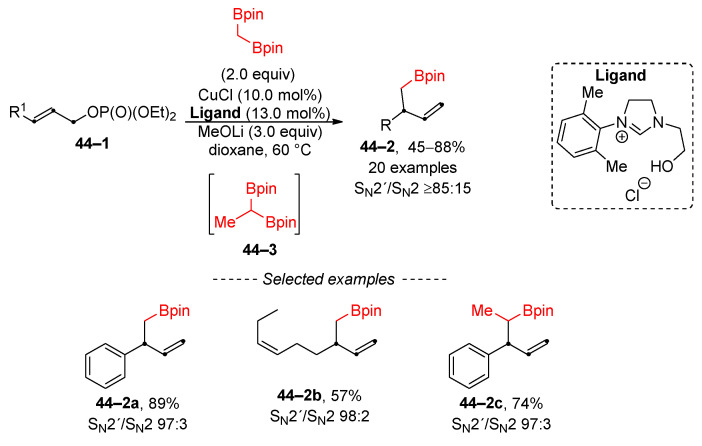
Gem-diborylalkanes in copper/NHC ligand-catalyzed allylic substitution.

Beyond boronic acids, copper-catalyzed allylic substitution has been successfully extended to include *gem*-diboryl alkanes (Scheme 44) [184]. This reaction catalyzed by an NHC ligand achieves an excellent S_N_2′/S_N_2 substitution ratio. In addition to *gem*-diborylmethane, the optimized reaction conditions allow for the use of *gem*-diborylethane **44**–**3,** as evidenced by the structure of the synthesized derivative **44**–**2c**. Similarly, Hoveyda and co-workers [185], have reported concurrent findings, employing a chiral ligand to synthesize enantiomerically pure products.

**Scheme 45:**
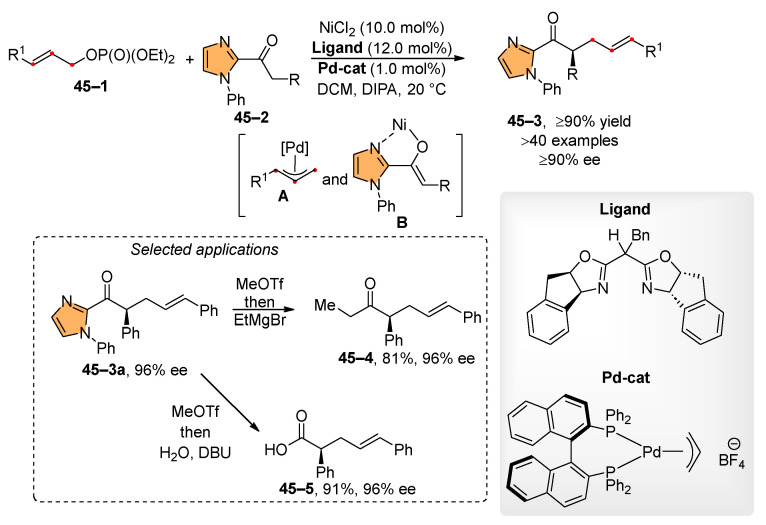
Palladium/nickel-catalyzed allylation of 2-acylimidazole.

Allyl phosphates are effective in the allylation of C-nucleophiles derived from carbonyl compounds. A typical example of this reaction is the asymmetric allylation of 2-acylimidazoles **45**–**2**, facilitated by Ni/Pd dual catalysis (Scheme 45) [186]. In this process, a nickel complex activates the imidazole derivative **45**–**2**, while a palladium catalyst converts the allyl phosphate **45**–**1** into a π-allyl complex **A**. The formed π-allyl complex **A** than alkylates the activated imidazole **B**. A significant feature of this work is the high number of synthesized compounds, along with the high isolated yields and high enantiomeric excess. Furthermore, the imidazole group can be readily activated through alkylation with methyl triflate. Subsequently, the hydrolysis of the activated imidazole produces acid **45**–**5**, while the reaction with ethylmagnesium bromide results in ketone **45**–**4**. In both cases, products **45**–**5** and **45**–**4** are obtained with the retention of the absolute configuration at the stereogenic center.

**Scheme 46:**
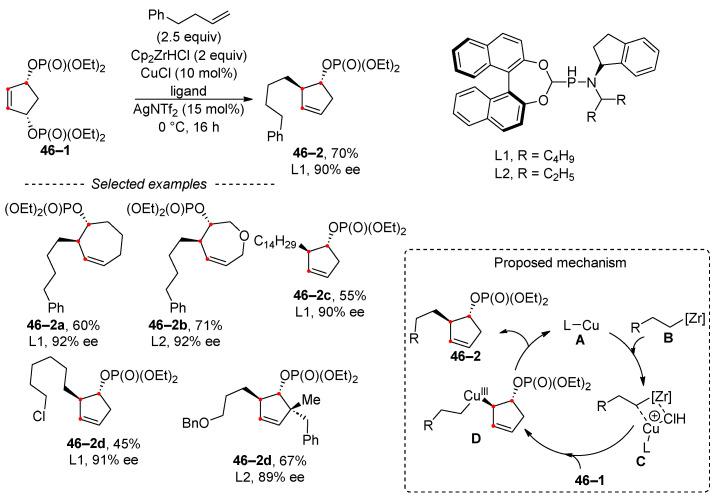
Copper-catalyzed desymmetrization of *meso*-bisphosphates.

The concept of transition-metal-catalyzed asymmetric allylic substitution was applied for the desymmetrization of *meso*-bisphosphates (Scheme 46) [187]. In this study, bisphosphates undergo monoselective alkylation leading solely to the formation of cycloalkenyl phosphates. The reaction was optimized using cyclopentenyl derivative **46**–**1,** giving the corresponding enantiomer **46**–**2a**. The method allows for the desymmetrization of both five- and seven-membered rings, yielding products with uniformly high enantiomeric purity, as shown in the selected examples. The authors postulated that AgNTf_2_ is pivotal in the generation of catalytic moiety **A** from copper chloride. The alkyl zirconium reagent, produced in situ from the Schwarz reagent and alkene, undergoes Zr–Cu transmetalation. Subsequent oxidative addition and reductive elimination steps form intermediate **D** and regenerate catalyst **A**, along with product **46**–**2**.

**Scheme 47:**
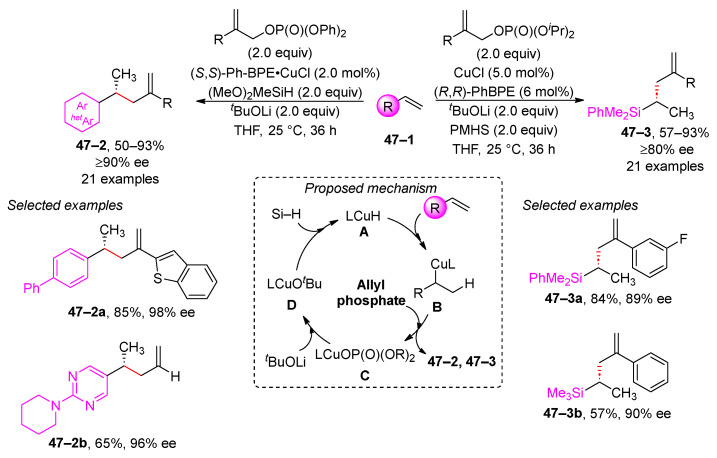
Copper–H-catalyzed hydroallylation of vinylarenes.

Allyl phosphates have been used in the Cu-H catalyzed hydroallylation of vinyl arenes and propene (Scheme 47) [188,189]. Both studies elucidate a similar mechanism. Initially, the reaction starts with the hydrocupration of the terminal double bond, followed by an enantioselective allylic substitution. This process results in the formation of complex **C**, which is then converted to complex **A** by a two-step substitution process, which involves the substitution of the phosphate ligand with *^t^*BuOLi followed by the introduction of the hydride ion. Each study employed different reducing agents. Buchwald selected dimethoxymethylsilane, while Xiong favored polymethylhydrosiloxane (PMHS). Furthermore, Buchwald used the (*S*,*S*)-Ph-BPE ligand [189], whereas Xiong used the enantiomeric (*R*,*R*)-Ph-BPE ligand [188]. In both cases, products **47**–**2** and **47**–**3** were obtained with high enantiomeric purity.

**Scheme 48:**
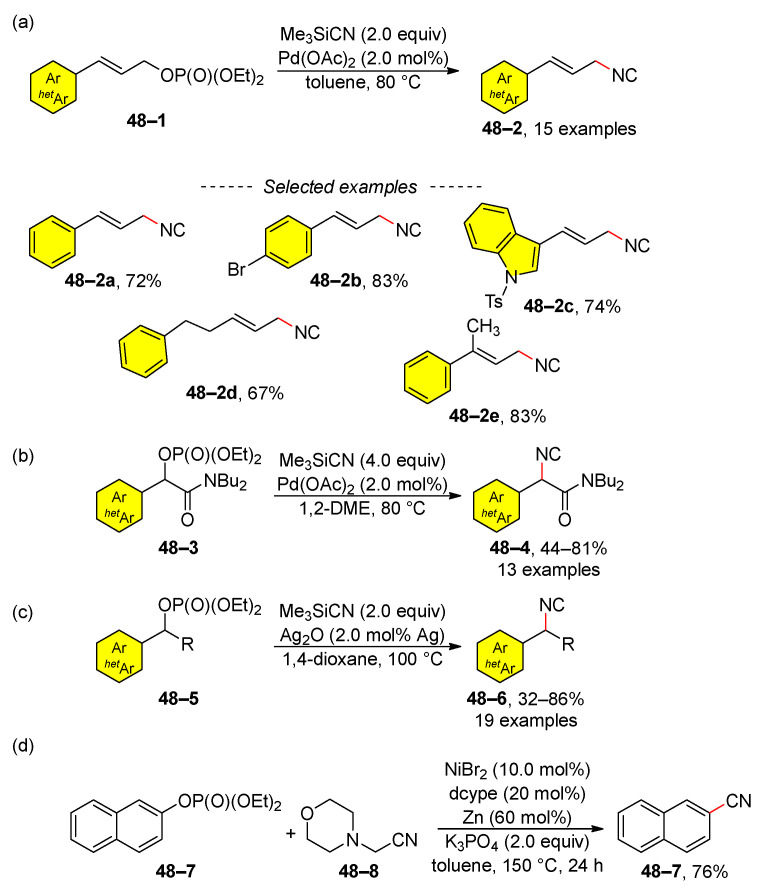
Transition-metal-catalyzed isocyanation of (**a**) allyl, (**b**,**c**) benzyl and (**d**) 2-naphthyl phosphates.

Recently, Okhuma published the synthesis of isonitriles based on a palladium-catalyzed reaction of allyl diethyl phosphates **48**–**1** with trimethylsilyl cyanide (Scheme 48a) [190]. The authors precisely optimized the course of the reaction and found that the use of allyl phosphate **48**–**1** is crucial for the successful formation of isonitriles **48**–**2**. In contrast, allyl acetates yielded only the corresponding nitriles. However, it is important to note that the reaction scope is somewhat limited and the tolerance for various functional groups is relatively low. Although a complete mechanism has not yet been fully elucidated, the authors propose that isocyanation is likely catalyzed by a Pd(II) complex rather than a Pd(0) complex. The same group extended the reaction scope of the isocyanation reaction to include benzyl diethyl phosphates (Scheme 48b,c) [191,192]. Yamaguchi’s group established conditions for the nickel-catalyzed cyanation of phenol derivatives (Scheme 48d) [193]. Although the study focused on aryl carbamates and pivalates, other substrates, such as 2-naphthyl tosylate, trifluorosulfonate, and diethyl phosphate **48**–**7**, were shown to be effectively used.

**Scheme 49:**
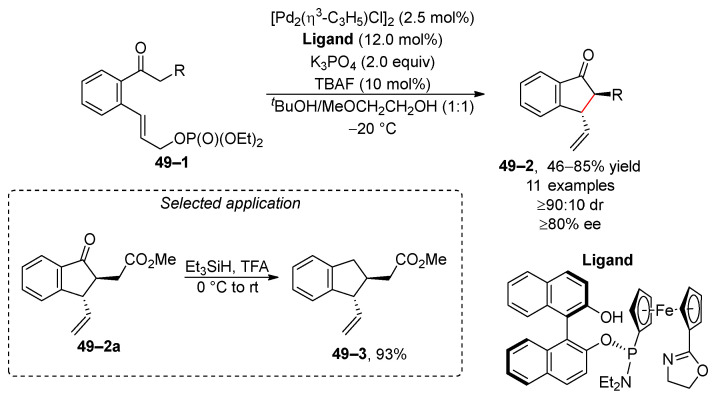
Palladium-catalyzed asymmetric cyclization of aromatic ketones.

An interesting variation of asymmetric allyl alkylation is the intramolecular approach. Hou and co-workers exploited palladium-catalyzed cyclization to transform 1,2-disubstituted benzenes **49**–**1** into benzocyclopentanones **49**–**2** (Scheme 49) [194]. The reaction typically yields products with high diastereomeric and enantiomeric purity, which makes the developed methodology attractive with respect to a wide range of natural compounds with the benzocyclopentanone motif. Furthermore, the selected benzocyclopentanone **49**–**2a** was reduced with triethylsilane in trifluoroacetic acid to benzocyclopentane **49**–**3** in an almost quantitative yield. Additionally, a novel approach based on palladium-isothiourea relay catalysis facilitated the allylation of α-amino acids using diethyl allyl phosphates [195].

**Scheme 50:**
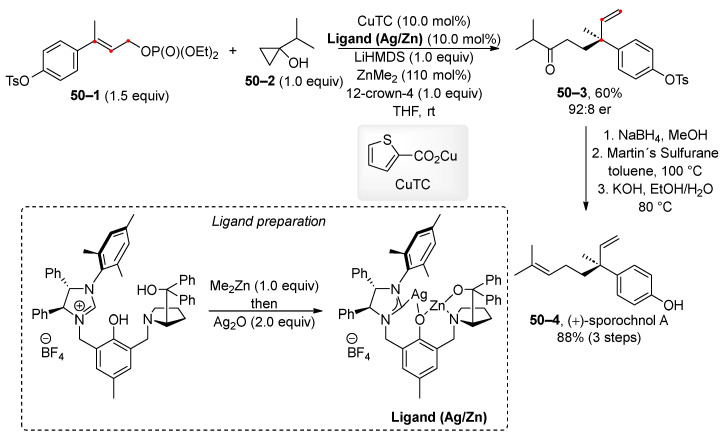
Total synthesis of (+)-sporochnol A.

Trost and co-workers developed an asymmetric allylic alkylation catalyzed by copper along with a bifunctional ligand. The bifunctional ligand combines NHC and prolinol moieties connected via a phenolic spacer. At the beginning of the reaction, the active ligand is synthesized in situ from the ligand (Ag/Zn) by its conversion to a Cu/Zn heterobimetallic complex. The developed method was successfully applied to the asymmetric synthesis of (+)-sporochnol A (**50**–**4**) (Scheme 50) [196].

**Scheme 51:**
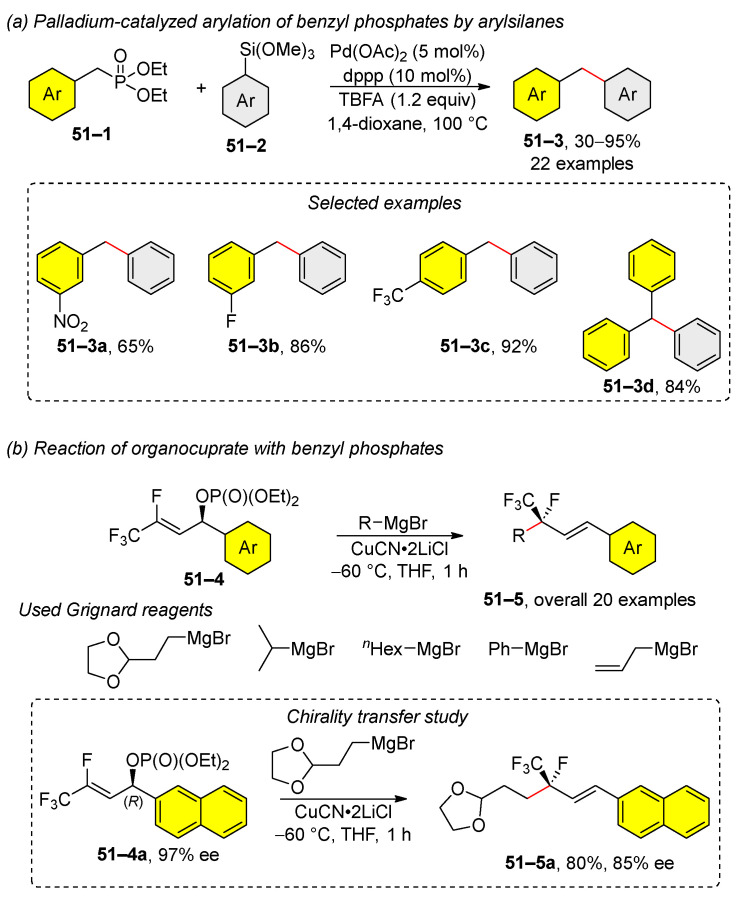
Reaction of benzyl phosphates with organometallic reagents.

Transition-metal-catalyzed reactions of benzyl phosphates with organometallic reagents were also studied. In the first report, simple arylsilanes were used to synthesize diarylmethanes (Scheme 51a) [197]. This reaction is characterized by high yields of diarylmethanes represented by a general structure **51**–**3**, although the reaction scope is somewhat limited to a few robust functional groups. In contrast, Koert and co-workers explored the reaction of benzyl-type organophosphate **51**–**4** with organocuprate (Scheme 51b) [198]. This reaction predominantly gives the γ-isomer **51**–**5** via S_N_2′ pathway, while the reaction scope is limited to organocuprates derived from Grignard reagents. Chirality transfer experiments were only partially successful, as evident from the synthesis of compound **51**–**5a**.

**Scheme 52:**
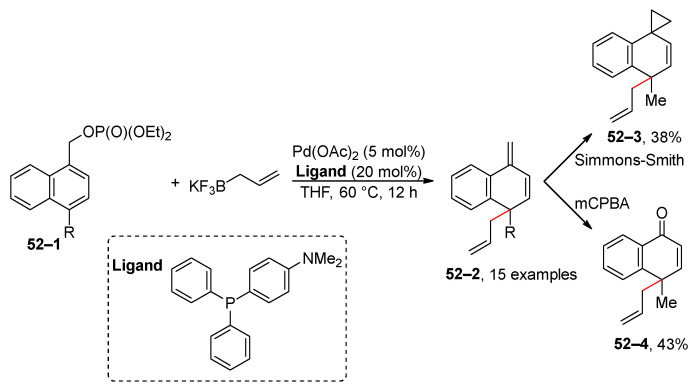
Dearomative allylation of benzyl phosphates.

Dearomative cross-coupling reactions have emerged as a crucial methodology for the preparation of partially aromatic or fully dearomatized arenes [199] and heteroaromatic compounds [200,201,202]. An illustrative case of this approach for benzyl phosphates is the transformation of phosphate **52**–**1** into dihydronaphthalenes **52**–**2** (Scheme 52) [203]. Products **52**–**2** are prone to isomerization when exposed to silica gel. In a selected example, it was demonstrated that further modification, such as the Simmons–Smith reaction or oxidation, can be successfully carried out. Additionally, the incorporation of a nitrile group at the benzyl position of the starting substance **52**–**1** improves the stability of the dihydronaphthalene products. However, this modification simultaneously reduces the reactivity of phosphate [204].

**Scheme 53:**
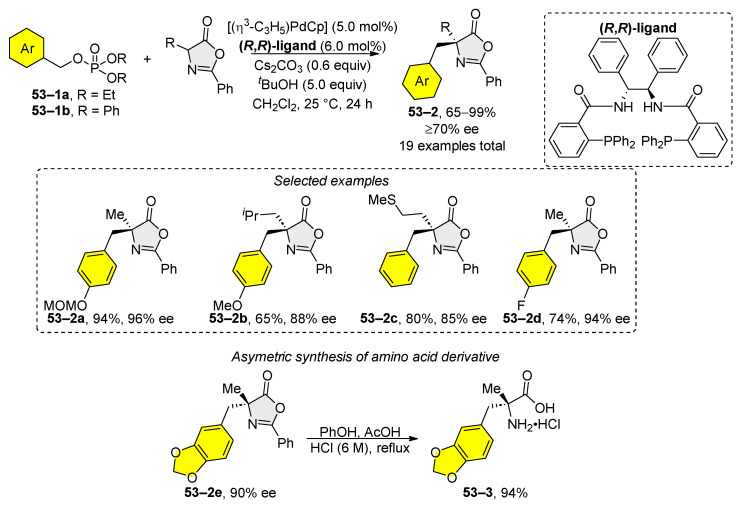
Palladium-catalyzed benzylation of carbonyl compounds en route to chiral amino acid derivatives.

Benzyl phosphates and carbonates have been used for the benzylation of azlactones (Scheme 53) [205]. Through comprehensive studies of reaction conditions, it was discovered that the (*R*,*R*)- or (*S*,*S*)-dppba ligands are highly effective for the asymmetric benzylation of starting phosphates **53**–**1a** and **53**–**1b**. The reaction outcome of this reaction depends on the structure of the electrophilic reagents. The electron-neutral aryl and heteroaryl carbonates of the benzyl type undergo alkylation without a base. However, phosphates **53**–**1a** and **53**–**1b** were alkylated in the presence of cesium carbonate, indicating that the carbonate group acts as a base during alkylation. The enantiomeric purity of the alkylation products was evaluated by the hydrolysis of azlactones to amino acid **53**–**3**. The authors also suggested a mechanism for the benzylation process that involves the corresponding η^3^-complex or a η^1^-complex, which then isomerizes to a η^3^-complex.

**Scheme 54:**
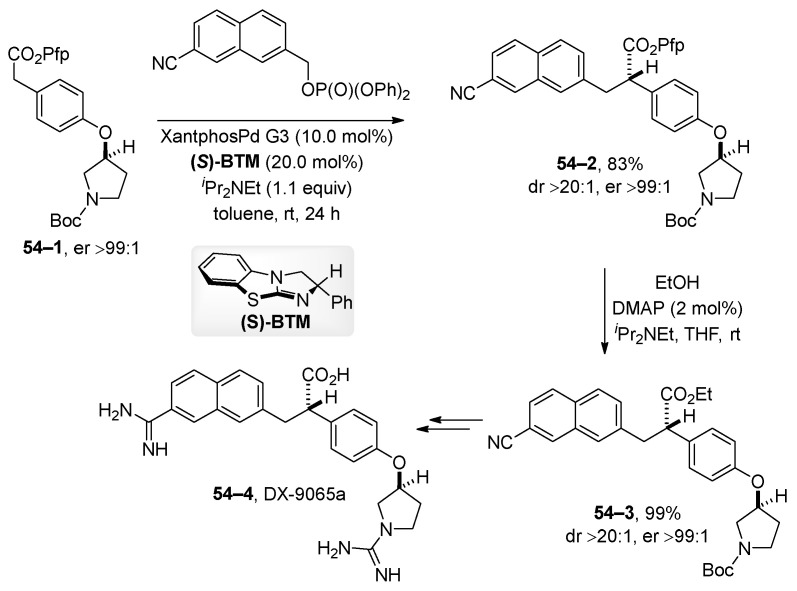
Enantioselective synthesis of the thrombin inhibitor DX-9065A by palladium-catalyzed benzylation.

The enantioselective palladium-catalyzed benzylation of carboxylic acid esters was achieved using the (*S*)-BTM ligand. The optimized reaction conditions were used for the formal synthesis of the thrombin inhibitor DX-9065A (Scheme 54) [206]. The starting pentafluorophenyl ester **54**–**1** was benzylated under optimized reaction conditions to give the enantiomerically pure product **54**–**2**. The isolated pentafluorophenyl ester **54**–**2** was then converted to ethyl ester **54**–**3** in a quantitative yield. Finally, the synthesis of the inhibitor DX-9065A can be accomplished by following a previously reported procedure [207].

**Scheme 55:**
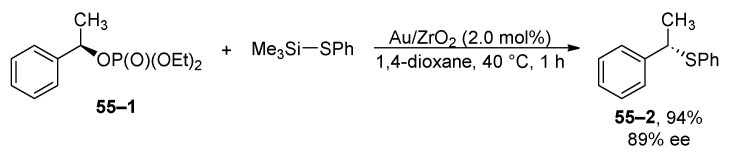
Gold-catalyzed enantioselective thioetherification of benzyl phosphate.

In 2022, the thioetherification of allyl, benzyl, and propargyl phosphates was published (Scheme 55) [208]. The reaction’s scope was exclusively evaluated using silylated thiophenol. It is crucial, however, to highlight that the reaction conditions were restricted to unfunctionalized phosphates. The complete inversion of the configuration in the chiral benzyl phosphate **55**–**1** suggests that the reaction mechanism involves the activation of both reactants by a heterogeneous catalyst, followed by an S_N_2 substitution process.

**Scheme 56:**
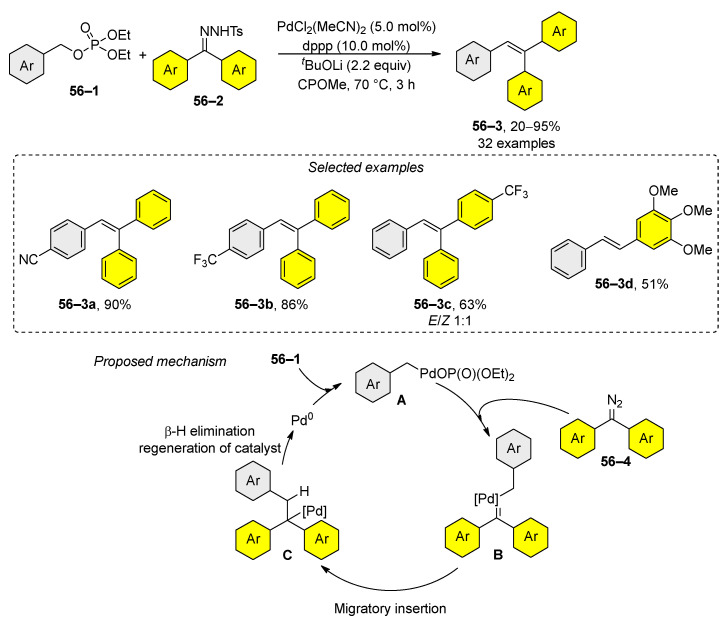
Palladium-catalyzed cross-coupling of *N*-tosylhydrazones with benzyl phosphates.

Recently, a novel synthesis of di- and trisubstituted alkenes from benzyl phosphates **56**–**1** and *N*-tosylhydrazones **56**–**2** was reported (Scheme 56) [209]. From a mechanistic perspective, two aspects are pivotal. Initially, the mechanism involves the oxidative addition of a palladium catalyst to create complex A, while concurrently, the hydrazone is converted in situ into diazo compound **56**–**4**. This compound subsequently interacts with complex **A** to form complex **B**. Finally, migratory insertion, followed by β-H elimination, yields the reaction product **56**–**3**. However, the method’s limitation lies in its applicability primarily to symmetric trisubstituted alkenes **56**–**3a** and **56**–**3b**. Stereoselective synthesis in this context is challenging because it results in a mixture of stereoisomers **56**–**3c**. In contrast, the formation of disubstituted alkene **56**–**3d** occurs exclusively as the (*E*) stereoisomer.

## 6. Oxidation and Reduction Reactions of Organophosphates

**Scheme 57:**
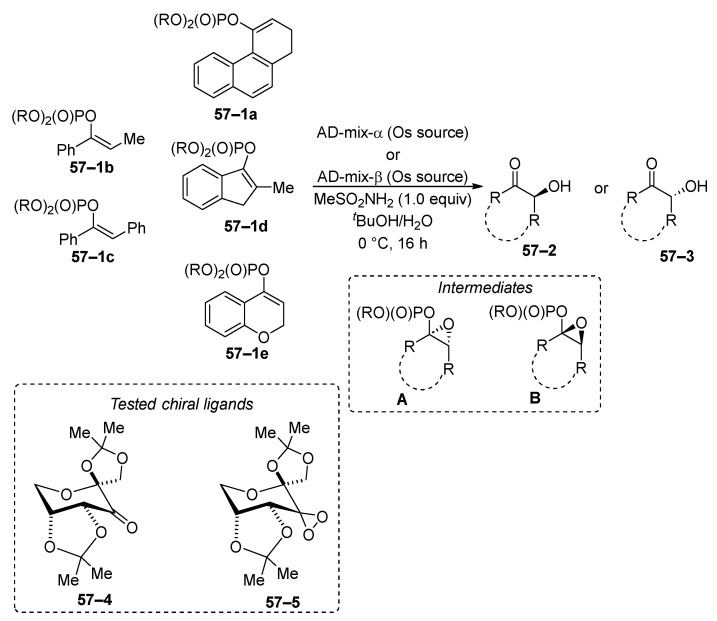
Enantioselective hydroxylation of cyclic enol phosphates.

Vinyl phosphates are amenable to modification via oxidation processes. Under osmium-catalyzed Sharpless epoxidation conditions [210], the oxidation of both acyclic and cyclic vinyl phosphates **57**–**1** leads to the formation of enantiomerically pure hydroxyketones **57**–**2** or **57**–**3** (Scheme 57) [211,212,213]. The efficacy of commercially available (DHQ)_2_PHAL and (DHQD)_2_PHAL ligands has been compared with ligands **57**–**4** and **57**–**5**. Epoxides **A** and **B** are common intermediates during the oxidation of vinyl phosphates, which are then hydrolyzed to yield hydroxyketones **57**–**2** and **57**–**3**. The observed enantiomeric excess for both (S)- and (R)-alcohols (**57**–**2** and **57**–**3**) ranges from 13% to 100%, across both commercially available and experimentally tested ligands. In a related study, Jones and co-workers explored the epoxidation and hydroxylation of cyclic vinyl phosphate acetate and trialkylsilyl ethers. They observed that vinyl phosphate and trialkylsilyl ethers performed significantly worse than the corresponding vinyl acetate [214].

**Scheme 58:**
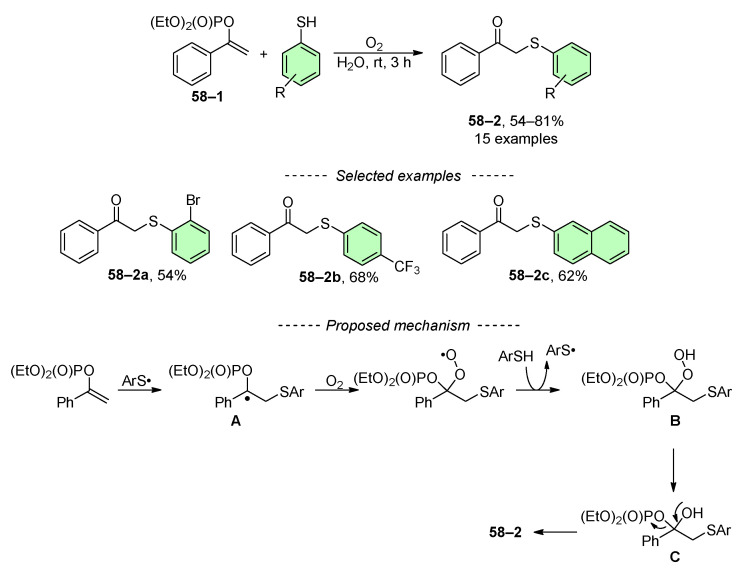
Transition-metal-free conversion of vinyl phosphates to β-keto sulfides.

As a continuation of their previous work [215], Lei and co-workers developed a method for the oxidative addition reaction of vinyl phosphates **58**–**1** to β-keto sulfides **58**–**2** (Scheme 58) [216]. This reaction is conducted in an oxygen-rich environment. The proposed mechanism begins with the generation of an S-radical, which interacts with the activated double bond in the vinyl phosphate to form intermediate **A**. Oxygen then reacts with thiophenol to produce hydroperoxide **B**, which is reduced to intermediate **C**. The final step is the elimination of (EtO)_2_(O)POH, resulting in the formation of sulfide **58**–**2**. It is crucial to recognize that the applicability of this reaction is limited to substrates featuring simple functional groups (Scheme 58).

**Scheme 59:**
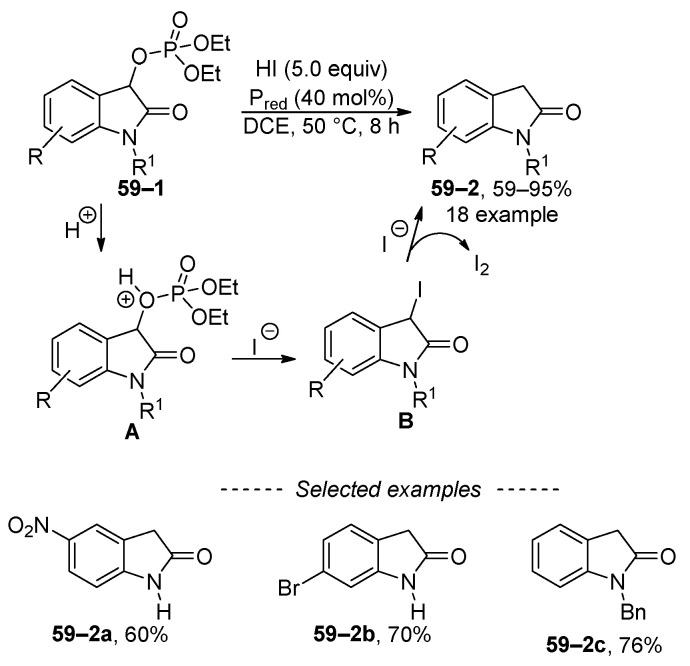
Synthesis of 2-oxindoles by reductive dephosphorylation of 2-oxindolyl phosphates.

Phosphates derived from 2-oxindoles can undergo reduction by means of hydroiodic acid (Scheme 59) [217]. This reduction process is initiated by the protonation of the phosphate group, leading to intermediate **A**. Subsequently, intermediate **A** is attacked by the iodide anion, resulting in the formation of 3-iodo indole derivative **B**. The reduction is completed by a subsequent substitution at the iodine atom. However, due to harsh reaction conditions, tolerance for functional groups is restricted to simple and rather robust groups, such as the nitro group in **59**–**2a** and halogen in **59**–**2b**.

**Scheme 60:**
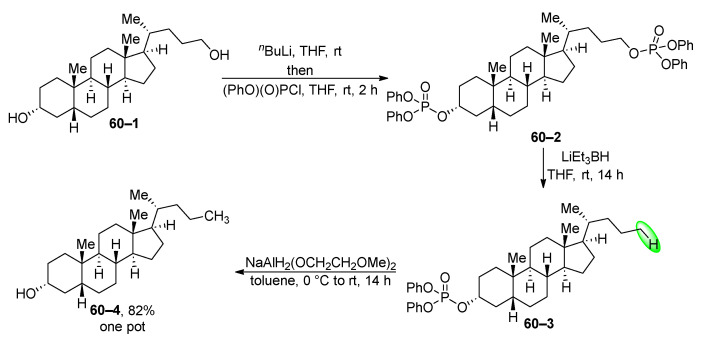
Chemoselective reduction of primary alkyl phosphate by lithium triethylborohydride.

Primary and secondary alkyl phosphates can be chemoselectively reduced with various hydrides (Scheme 60) [218]. The authors employed a chemoselective reduction strategy targeting a phosphate **602** derived from lithhocholanyl alcohol **60**–**1** in a one-pot setup. This phosphate **60**–**2** was then reduced to monophosphate **60**–**3** by treatment with lithium triethylborohydride. Finally, the diphenyl phosphate group was removed by treatment with sodium bis(2-methoxyethoxy)aluminum hydride.

**Scheme 61:**
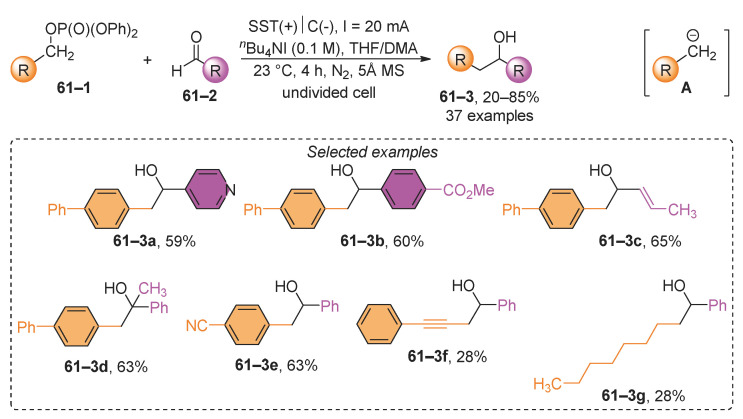
Radical deoxygenative coupling of benzyl phosphates with aldehydes and ketones.

Electrochemical synthesis offers another approach for organophosphate reduction to secondary alcohols from diphenyl benzyl phosphates **61**–**1** (Scheme 61) [219]. This reduction is carried out in an undivided cell equipped with a stainless steel (SST) anode and a graphite cathode, all at room temperature. A critical step in this synthesis is the reduction of phosphate **61**–**1** to anion **A**, which then reacts with a carbonyl group. The reduction potential for the phosphate group is established at 2.8 V via cyclic voltammetry. The optimized conditions are versatile, supporting a wide array of functional groups. Beyond aldehydes, this method has been successfully applied to ketones **61**–**3d,** alkyl **61**–**3g,** and propargyl **61**–**3f** phosphates. In addition, Morzycki reported the electrochemical cholesterylation of cholesteryl diphenyl phosphate [220].

## 7. Conclusions

In this review, we have highlighted the significant aspects of organophosphates and their applications in organic synthesis. Organophosphates are primarily utilized in cross-coupling reactions for C–C-bond formation, particularly in transition-metal-catalyzed allyl substitutions. Additionally, the oxidation and reduction of vinyl and alkyl phosphates have become increasingly relevant. Although not explicitly discussed in this review, organophosphates’ role in organic synthesis, particularly in competition with alternative electrophiles featuring activated C–O bonds such as tosylates, acetates, and carbonates, merits attention. In specific instances, organophosphates may outperform these alternatives in stereoselective synthesis, notably in the formation of tetrasubstituted alkenes. However, this advantage is often challenged by the fact that acetates and tosylates frequently yield comparable results. Tosylates or acetates hold an edge due to their simpler ^1^H and ^13^C NMR spectra, in addition to the cost effectiveness and availability of their precursors. In addition, it is important to note the regulatory considerations surrounding the use of many organophosphorus compounds, given their documentation in the context of chemical-weapons conventions.

However, there are notable limitations in the area of multicomponent reactions involving organophosphates. This issue stems from the fact that organophosphates possess three distinct carboneous substituents. Regrettably, in most reactions, only one of these groups is typically utilized, which considerably restricts the atom economy of organophosphates.

The distinctive structure and properties of the phosphate group pave the way for innovative approaches in sustainable chemistry, particularly considering the natural abundance of phosphates. Currently, the potential of organophosphate applications in organic synthesis, such as their electrochemical transformations, remains largely untapped. Prospective advancements in organophosphate chemistry involve catalytic reactions in which phosphate electrophiles are formed in a catalytic manner.
